# MaHPIC malaria systems biology data from *Plasmodium cynomolgi* sporozoite longitudinal infections in macaques

**DOI:** 10.1038/s41597-022-01755-y

**Published:** 2022-11-24

**Authors:** Jeremy D. DeBarry, Mustafa V. Nural, Suman B. Pakala, Vishal Nayak, Susanne Warrenfeltz, Jay Humphrey, Stacey A. Lapp, Monica Cabrera-Mora, Cristiana F. A. Brito, Jianlin Jiang, Celia L. Saney, Allison Hankus, Hannah M. Stealey, Megan B. DeBarry, Nicolas Lackman, Noah Legall, Kevin Lee, Yan Tang, Anuj Gupta, Elizabeth D. Trippe, Robert R. Bridger, Daniel Brent Weatherly, Mariko S. Peterson, Xuntian Jiang, ViLinh Tran, Karan Uppal, Luis L. Fonseca, Chester J. Joyner, Ebru Karpuzoglu, Regina J. Cordy, Esmeralda V. S. Meyer, Lance L. Wells, Daniel S. Ory, F. Eun-Hyung Lee, Rabindra Tirouvanziam, Juan B. Gutiérrez, Chris Ibegbu, Tracey J. Lamb, Jan Pohl, Sarah T. Pruett, Dean P. Jones, Mark P. Styczynski, Eberhard O. Voit, Alberto Moreno, Mary R. Galinski, Jessica C. Kissinger

**Affiliations:** 1 Institute of Bioinformatics, University of Georgia, Athens, GA 30602 USA; 2 Center for Tropical and Emerging Global Diseases, University of Georgia, Athens, GA 30602 USA; 3 Emory Vaccine Center, Yerkes/Emory National Primate Research Center, Emory University, Atlanta, GA 30329 USA; 4 Center for Integrative Genomics, School of Biology, Georgia Institute of Technology, Atlanta, GA 30332 USA; 5 School of Chemical & Biomolecular Engineering, Georgia Institute of Technology, Atlanta, GA 30332 USA; 6 The Wallace H. Coulter Department of Biomedical Engineering, Georgia Institute of Technology and Emory University, Atlanta, GA 30332 USA; 7 Complex Carbohydrate Research Center, Department of Biochemistry, University of Georgia, Athens, GA 30602 USA; 8 Division of Endocrinology, Metabolism & Lipid Research, Washington University School of Medicine, St. Louis, MO 63110 USA; 9 Division of Pulmonary, Allergy, Critical Care, & Sleep Medicine, Department of Medicine, Emory University School of Medicine, Atlanta, GA 30322 USA; 10 Lowance Center for Human Immunology, Emory University, Atlanta, GA 30322 USA; 11 Department of Pediatrics, Emory University School of Medicine, Atlanta, GA 30322 USA; 12 Department of Mathematics, Institute of Bioinformatics, University of Georgia, Athens, GA 30602 USA; 13 Division of Infectious Diseases, Department of Medicine, Emory University School of Medicine, Atlanta, GA 30322 USA; 14 Yerkes/Emory National Primate Research Center, Emory University, Atlanta, GA 30329 USA; 15 Biotechnology Core Facility Branch, Centers for Disease Control and Prevention, Atlanta, GA 30333 USA; 16 Department of Genetics, University of Georgia, Athens, GA 30602 USA; 17 Present Address: Department of Medicine, Vanderbilt University Medical Center, Nashville, TN 37232 USA; 18 Present Address: Cancer Data Science Initiatives, Frederick National Laboratory for Cancer Research, Post Office Box B, Frederick, MD 21702 USA; 19 Present Address: Center for Tropical and Emerging Global Diseases, University of Georgia, Athens, GA 30602 USA; 20 Present Address: Department of Pediatrics, Emory University School of Medicine, Atlanta, GA 30322 USA; 21 Present Address: Division of Pulmonary, Allergy, Critical Care, and Sleep Medicine, Department of Medicine, Emory University School of Medicine, Atlanta, GA 30322 USA; 22 Present Address: Laboratório de Malária, Instituto René Rachou/Fiocruz Minas, Av. Augusto de Lima 1715, Belo Horizonte, MG 30190 009 Brazil; 23 Present Address: Center for Vaccines and Immunology, University of Georgia, Athens, GA 30605 USA; 24 Present Address: Senior Public Health Informaticist, MITRE Corp, Atlanta, GA 30345 USA; 25 Present Address: Department of Biomedical Engineering, University of Texas at Austin, Austin, TX 78712 USA; 26 Present Address: Interdisciplinary Disease Ecology Across Scales Research Traineeship Program, Institute of Bioinformatics, Center for the Ecology of Infectious Diseases, University of Georgia, Athens, GA 30602 USA; 27 Present Address: Department of Medicine, Brigham and Women’s Hospital, Harvard Medical School, Boston, MA 02115 USA; 28 Present Address: Valted Seq, 704 Quince Orchard Rd, Gaithersburg, MD 20878 USA; 29 Present Address: Federal Drug Administration, Silver Spring, MD 20993 USA; 30 Present Address: Division of Pulmonary, Critical Care, and Sleep Medicine, Department of Medicine, University of Florida, Gainesville, FL 32603 USA; 31 Present Address: Center for Tropical and Emerging Global Disease, University of Georgia, Athens, GA 30602 USA; 32 Present Address: Center for Vaccines and Immunology, Department of Infectious Diseases, College of Veterinary Medicine, University of Georgia, Athens, GA 30602 USA; 33 Present Address: Department of Biosciences and Diagnostic Imaging, College of Veterinary Medicine, University of Georgia, Athens, GA 30602 USA; 34 Present Address: Department of Biology, Wake Forest University, Winston Salem, NC 27103 USA; 35 Present Address: Institutional Animal Care and Use Committee, Research Compliance and Research Integrity Office, Emory University, Atlanta, GA 30322 USA; 36 Present Address: Casma Therapeutics, Cambridge, MA 02139 USA; 37 Present Address: University of Texas at San Antonio, San Antonio, TX 78249 USA; 38 Present Address: Department of Pathology, University of Utah, Salt Lake City, UT 84112 USA; 39 Present Address: University of Tennessee, Knoxville, TN 37996 USA

**Keywords:** Malaria, Signs and symptoms, Adaptive immunity, Molecular fluctuations

## Abstract

*Plasmodium cynomolgi* causes zoonotic malarial infections in Southeast Asia and this parasite species is important as a model for *Plasmodium vivax* and *Plasmodium ovale*. Each of these species produces hypnozoites in the liver, which can cause relapsing infections in the blood. Here we present methods and data generated from iterative longitudinal systems biology infection experiments designed and performed by the Malaria Host-Pathogen Interaction Center (MaHPIC) to delve deeper into the biology, pathogenesis, and immune responses of *P. cynomolgi* in the *Macaca mulatta* host. Infections were initiated by sporozoite inoculation. Blood and bone marrow samples were collected at defined timepoints for biological and computational experiments and integrative analyses revolving around primary illness, relapse illness, and subsequent disease and immune response patterns. Parasitological, clinical, haematological, immune response, and -omic datasets (transcriptomics, proteomics, metabolomics, and lipidomics) including metadata and computational results have been deposited in public repositories. The scope and depth of these datasets are unprecedented in studies of malaria, and they are projected to be a F.A.I.R., reliable data resource for decades.

## Abstract (summary)

**Table:** 

Measurement(s)	parasitemia • blood Chemistry • reticulocyte count • platelet activation status • neutrophil and monocyte activation after stimulation • T-cell phenotyping • B-cell phenotyping • monocyte phenotyping • dendritic cell phenotyping • lymphocyte phenotyping • multiplexed cytokine assay • erythropoietin measurement • total IgG concentration • transcriptome • metabolomics • lipidomics • proteomics
Technology Type(s)	brightfield microscopy • iSTAT machine • immunological flow cytometry • chemokine receptor markers • intracellular staining • ELISA • LC/MS
Factor Type(s)	Parasitemia
Sample Characteristic - Organism	Macaca mulatta • Plasmodium cynomolgi B/M strain • Plasmodium cynomolgi Ceylon
Sample Characteristic - Environment	venous blood • capillary blood

## Background & Summary

The Malaria Host-Pathogen Interaction Center (MaHPIC) posed the overarching hypothesis that “Nonhuman Primate host interactions with *Plasmodium* pathogens as model systems would provide insights into mechanisms, as well as indicators for, human malarial disease conditions^1^. From September 2012 through September 2017, highly collaborative MaHPIC teams of transdisciplinary scientists used systems biology approaches to design and implement 32 experiments named E01 – E32, including 12 longitudinal *in vivo* parasite infection experiments involving nonhuman primates (NHPs) to study molecular details that underpin pathogenesis and immunity: how malaria parasites cause disease and how infected hosts recover. The MaHPIC’s data and findings are pertinent to understanding malaria caused by five species of *Plasmodium* parasites: *Plasmodium falciparum*, *P. vivax*, *P. ovale*, *P. malariae*, and two zoonotic species, *P. knowlesi* and *P. cynomolgi* (reviewed in^2–5^).

**Fig. 1:**
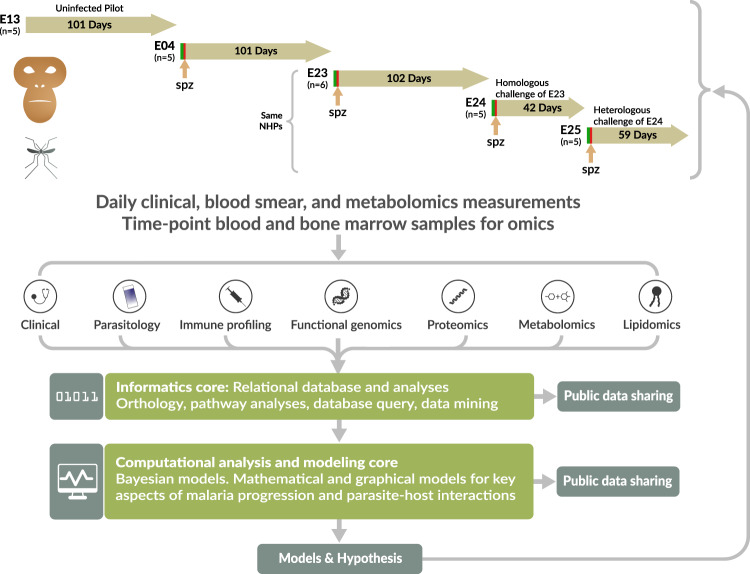
Iterative *Plasmodium cynomolgi* infections in *Macaca mulatta*. The experimental approach was designed to (1) use *P. cynomolgi* in a *M. mulatta* non-human primate host to discover the characteristics of a relapsing infection as a model for the human pathogen *P. vivax* and (2) explore the dynamics of the host response to infection. Host samples, their associated metadata and resulting experimental data received unique identifiers that were tracked in a LIMS. Raw and analyzed experimental results and associated metadata were validated and made available internally in a relational database for internal use. The depicted process was iterative, and relied upon clinicians, veterinarians, systems biologists, multiple omics experts, data management and informatics experts, malariologists, immunologists, and mathematical modelers to follow the results and generate new models of disease and hypotheses for further validation. All results were deposited in public repositories. Spz – sporozoite inoculation; NHP – nonhuman primates; n – number of NHPs in each experiment. Experiments 23, 24 and 25 utilized the same *M. mulatta* animals and either the same (homologous) or different (heterologous) *P. cynomolgi* strain.

Here, we showcase MaHPIC experiments involving infections of *Macaca mulatta* (rhesus monkeys) initiated with *Plasmodium cynomolgi* sporozoites (Fig. 1). This species, like *P. vivax* and *P. ovale*, develops dormant forms in the liver, called hypnozoites, which can become activated to cause relapsing infections in the blood^6,7^. The MaHPIC experiments were designed to study the primary blood-stage infections and subsequent relapses, while detailing clinical, haematological, parasitological, immunological, transcriptomic and metabolomic features to investigate the overall progression and resolution of disease^1^. In addition, blood samples were allocated for complementary lipidomic and proteomic analyses.

Experiment 13 was implemented to test-drive all technical and data collection procedures in the presence of drug treatment but the absence of infection^8^. Experiments 04, 23, 24, and 25 involved baseline analyses, *P. cynomolgi* sporozoite infections, and specific timepoint (TP) sample collections for up to 100 days (Fig. 1). The experiment numbers reflect their designation in the MaHPIC’s Laboratory Information Management System (LIMS). Figures 3–6 show projected ‘Idealized’ experiments and ‘Actual’ data profiles. Antimalarial drug treatments were provided as required to subcuratively treat the animals – in efforts to prevent severe illness, to curatively treat the blood-stage infections, and to curatively treat the liver- and blood-stage infections at the end of each experiment. The MaHPIC routinely performed clinical blood chemistry (iSTAT tests), complete blood counts (CBCs), and blood smear readings to record parasitaemias. Blood and BM samples acquired at TPs were used for omic testing and immunological based experiments. As the infection experiments were iterative, the specific sampling and testing strategies were not identical for each experiment. Rather, each reflects adjustments made as incoming data were analysed and hypotheses and strategies were honed.

The MaHPIC produced >100,000 technical data files from these experiments and extensive metadata (e.g., on technical details, equipment, animal care, etc.), as well as mathematical models and analysis algorithms. The resulting collection of data and metadata for each experiment is referred to as a ‘dataset’, and each dataset was validated using standardized quality control procedures before being released to the MaHPIC team for internal analysis and subsequently to the public^9^. For E23, E24, and E25, samples were also acquired for microbiome analyses and the integration of such data with other datatypes to understand host-parasite-microbiome relationships (unpublished data). Each public dataset includes the origin of physical specimens (all barcoded), relationships to parent and child aliquots, standard operating procedures (SOPs), protocols detailing sample handling, processing, and storage, and analytical information on data production and quantitative *in silico* results. Updates to procedures and possible confounding factors are also indicated, such as a machine failure or changes in equipment or reagents. Each dataset was quality controlled and any nuances, for example, relating to specific clinical outcomes, sample generation, or data transfer were systematically catalogued in ‘README’ files.

The MaHPIC’s datasets offer unprecedented opportunities for studying molecular details in the course of experimental *Plasmodium*-NHP infections. There are frequent examples in the literature of clinical cross-sectional analyses, typically from symptomatic patient blood samples, and NHP infection studies addressing specific questions, but not using systems biological approaches. The MaHPIC’s datasets represent changes in host-parasite interactions during infection, and resulting altered biological, biochemical, and immunological pathways, which together provide insights into disease progression and recovery, and possibly new targets of intervention, including host-directed therapies or adjunctive therapies. The MaHPIC has analysed, integrated, and published results with selected datasets to test specific hypotheses on host-parasite dynamics and biological systems^8,10–15^. Terabytes of data remain to be explored and integrated by the research community now with current tools, and over time, with new, yet to be developed tools.

## Methods

### Animal use and approvals

Experiments involving NHPs were performed at the Yerkes National Primate Research Center (YNPRC), an AAALAC International-accredited facility, following ARRIVE guidelines and recommendations^16^. All experimental designs and protocols involving the NHPs were reviewed and approved by the Emory University Institutional Animal Care and Use Committee (IACUC): PROTO201700484-YER-2003344-ENTRPR-A. The NHPs used in these experiments were born, bred, and housed at the YNPRC. Male monkeys were assigned to eliminate confounding factors with anaemia due to menstruation. The *M. mulatta* subjects assigned typically ranged from 5–12 kg, and blood and BM sampling were restricted to 10 mg/kg/month, or 6 mg/kg/month if the animals become anaemic. All animals were socially housed, with 12-h light-dark cycles, and all conditions were compliant with the Animal Welfare Act and the Guide for the Care and Use of Laboratory Animals. Environmental enrichment, food, and physical manipulanda were provided daily. The animals received positive reinforcement training to habituate them to ear-stick blood collections for blood smear preparations and clinical tests requiring less than 150 µl. The animals were anaesthetised for blood draws using ketamine and/or Telazol and euthanised in one case due to severe illness^11^ via intravenous administration of barbiturates. This is an acceptable method of euthanasia for NHPs per the recommendations of the “AVMA Guidelines for the Euthanasia of Animals^17^”.

### Experimental design overview

The methods for each longitudinal NHP experiment (E13, E04, E23, E24 and E25) are divided below into two sections: 1) The experimental design and biological sample collection schemes for various data types, and 2) The detailed methods for the processing and analysis of each of the samples and data types. Sample types used in this manuscript are as follows: Bone Marrow (BM), Cryopreserved Plasma from LymphoPrep (CR), Peripheral Blood Mononuclear Cells (PB), Plasma (PS), Platelet (PL), Platelet from Capillary Samples (PC), Red Blood Cell Membrane (MN), Whole Blood (WB), and Whole Blood Capillary (WC). Note that the origins on plasma samples, from capillary or venous samples, is indicated throughout.

Experimental sample collection TPs were planned based on the projected progression of specific infections and questions being posed, and within ethically permitted and IACUC-approved time frames for acquiring designated volumes of blood and BM samples. The rhesus subjects ranged from 5 kg −12 kg, and blood and BM sampling were restricted to 10 mg/kg/month, or 6 mg/kg/month if the animals became anaemic. TPs were planned to address overarching or specific hypotheses and achieve designated goals. As expected, because of individual variations in the clinical progression of infection in individual NHPs, the clinically defined stage of infection sometimes differed between members of a cohort at the same TP. For this reason, it is important for anyone reusing these data to be aware of any differences between the projected or ‘Idealized’ and the ‘Actual’ disease progression for each NHP in each cohort and the corresponding clinical stage of each NHP at each TP when biological samples and supporting clinical information were collected. All biological parent samples and child aliquots were barcoded and tracked in a Nautilus Laboratory Information Management System (LIMS), developed specifically by the MaHPIC. Because sample volumes were restricted, the samples collected were aliquoted for distribution to the clinical, immune profiling, and omics analysis teams (aka cores in publicly deposited datasets and throughout this work). In the sample count descriptions below, the counts are for the number of samples used for measurements by those cores. Thus, the total number of analysis results are much higher than the total number of actual samples that were collected from the NHP subjects. Some datasets include control samples from an uninfected *M. mulatta*, with the identification code REe6.

### Experiment 13 (E13) Uninfected *M. mulatta* exposed to pyrimethamine to produce clinical, haematological, and omics control measures

#### E13 Experimental description

**Fig. 2:**
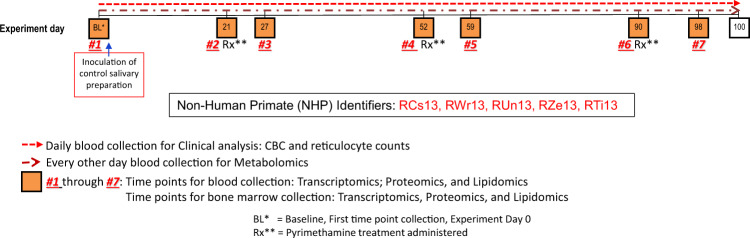
E13 Experimental timeline. The daily and time point sample collections and the administration of antimalarial medication is shown.

Experiment 13 involved malaria-naive *M. mulatta* (n = 5), approximately two years of age (IDs: RCs13, RWr13, RUn13, RZe13, RTi13), which were studied from May 28^th^, 2013 to September 5^th^, 2013. The experimental timeline is presented in Fig. 2 and in the supporting clinical information^8^. The animals were inoculated intravenously with a non-infectious mock preparation of salivary gland material derived from non-infected *Anopheles dirus* and then profiled for clinical, haematological, transcriptomic, metabolomic, and lipidomic measurements. TP and capillary blood samples were generated and analysed in this pilot study to establish logistics for subsequent experiments (E04, E23, E24, and E25) and to investigate the effects of the mock inoculation and anti-malarial drug pyrimethamine on normal individuals. E13 was designed for 100 days, with pyrimethamine administered at three different TPs to coincide with possible treatment days for experimentally infected rhesus macaques. Capillary blood samples were collected daily for the measurement of CBCs and reticulocytes. Additional capillary blood sample volume was collected every other day to obtain plasma for metabolomic analysis. WB samples and BM aspirates were collected at seven TPs before and after three rounds of drug administration for transcriptomic, proteomic, and lipidomic analyses. During the 100-day experiment, pyrimethamine was delivered (1 mg/kg) intramuscularly once on day 20, and for three successive days starting at days 52 and 90 (corresponding to TPs 2, 4, and 6), corresponding to predicted periods for sub-curative and curative experimental treatment regimens for *Plasmodium* infection of macaques. A summary of samples per NHP is available in Supplementary Table 1.

Briefly, the TPs collected are defined as:

- TP1 - Baseline, Pre-Rx control for each NHP.
- TP2 - Pyrimethamine was administered at 1 mg/kg IM (single dose).
- TP3 - Observations on NHP responses six days after administration of Pyrimethamine.
- TP4 - Pyrimethamine was administered at 1 mg/kg/day IM for three days.
- TP5 - Observations on NHP responses seven days after initiation of the anti-malarial regimen.
- TP6 - Pyrimethamine was administered at 1 mg/kg/day IM for three days.
- TP7 - Observations on NHP responses eight days after initiation of the anti-malarial regimen.

E13 Data types and sample counts

*E13 Clinical, haematological & parasitological sample counts (503 total)*

Daily WC were used for several clinical analyses. CBCs, reticulocyte counts, and blood smear slides to determine parasitaemias were generated. Blood chemistries were also performed on TP days. The following samples were analysed:

- 5 NHPs * 101 days * 1 specimen type (WC) = 503

*E13 Transcriptomics sample counts (70 total)*

The following samples were analysed:

- 5 NHPs * 7 TPs * 2 sample types (WB, BM) = 70

*E13 Metabolomics sample counts (26 total)*

Reference NHP samples, metabolomics standards and the following capillary blood samples were analysed:

- 5 NHPs * 5 TPs * 1 specimen type (PS) + 1 reference sample = 26

*E13 Lipidomics sample counts (20 total)*

Multiple targeted lipid classes (see methods) were quantified. The following samples were analysed:

- 5 NHPs * 4 TPs * 1 specimen type (WB) = 20

### Experiment 04 (E04): *M. mulatta* infected with *Plasmodium cynomolgi* M/B strain to produce and integrate clinical, haematological, parasitological, and omics measures of acute primary infection and relapses

#### E04 Experimental Description

Malaria-naive *M. mulatta* (n = 5) (IDs: RFa14, RIc14, RFv13, RMe14, RSb14), approximately three years of age, were studied from September 4^th^, 2013 to December 14^th^, 2013^8,10–12,14,15^. On September 5^th^, after baseline blood and BM sampling and testing, these NHPs were inoculated intravenously with about 2,000 *P. cynomolgi* M/B strain sporozoites, which had been produced and isolated at the Centers for Disease Control and Prevention (CDC) from the salivary glands of three *Anopheles* species (*An. dirus, An. gambiae*, and *An. stephensi*). The experiment was designed for 100 days, plus pre- and post- inoculation periods to allow time for preparing subjects for this experimentation (i.e., for the animal’s adjustment to the testing rooms and training for ear-stick procedures) and to administer curative treatments, respectively. The anti-malarial drug artemether was subcuratively administered (1 mg/kg) to three subjects (RMe14, RFa14, and RFv13) during their primary parasitaemia phase of infection in efforts to ward off possible severe clinical complications. Still, one of these subjects (RFv13) required euthanasia due to severe untreatable clinical complications as a result of hyperparasitemia^11^. After the primary parasitaemic phase, and relapses that followed (3), all animals received curative blood-stage treatment with artemether (4 mg/kg on the first day of treatment and 2 mg/kg/day for 7 days thereafter) to allow subsequent detection of bona fide relapse parasitaemias, initiated from the activation of hypnozoite forms of the parasite in the liver. At the end of the experiment, curative doses of primaquine (1 mg/kg/day for 7 days administered orally) and chloroquine (15 mg/kg/day for 3 days administered intramuscularly) were administered to all remaining subjects to eliminate the liver- and blood-stage infections, respectively.

The animals were profiled for clinical, haematological, parasitological, immunological, transcriptomic, metabolomic, lipidomic and proteomic measurements. Capillary blood samples were collected daily for the measurement of CBCs, reticulocytes, and parasitaemias. Additional capillary blood sample volume was collected every other day to obtain plasma (PS) for metabolomic analysis. In addition to capillary samples, venous blood and BM samples were collected at seven TPs for immunological, transcriptomic, proteomic, and lipidomic analyses. Additional transcriptomics results known as ‘E04R’ represent a ‘resequencing’ of the venous whole blood (WB) samples from E04, processed with updated SOPs and technology consistent with E23R, E24, and E25 (described below) so that results from all experiments are technically comparable. This adjustment also allowed for better detection of parasite transcripts, in addition to host transcripts.

**Fig. 3:**
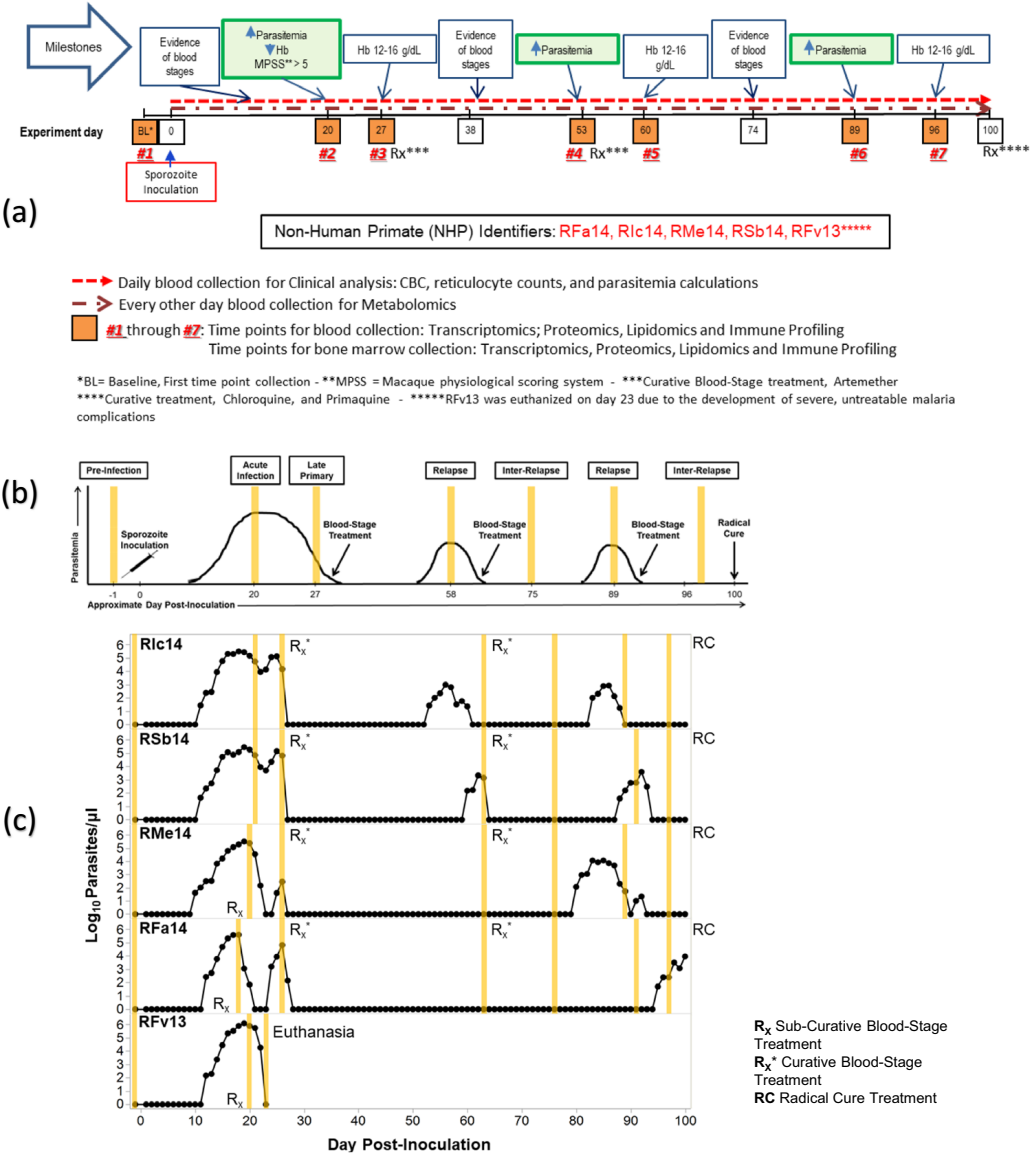
E04 projected ‘idealized’ and “actual’ experimental timeline and TP sample collection. (**a**) Timeline used to plan the experiment; (**b**) Figure showing Idealized clinical progression for the infected NHP cohort, and (**c**) Figure showing actual clinical progression that occurred during the experiment for each NHP in the cohort. b and c are reproduced^10^ with changes and permission from the authors under the Creative Commons license CC BY 4.0.

Sample collection TPs and a representation of both the Idealized and Actual clinical progression of infection are included in Fig. 3 and Table 1 and described in^10^. A summary of samples per NHP is available in Supplementary Table 2. Briefly:

- TP1 - Baseline (Pre-Infection): Uninfected control data for each NHP.
- TP2 - Acute Infection: Peak of infection determined by clinical, haematological, and parasitological assessment for each NHP. This TP is intended to characterise the host immune response and capture multi-omics data during the peak of parasitaemias when the NHPs are experiencing clinical signs of disease that may be severe or non-severe.
- The NHPs were monitored daily, and supporting clinical interventions were performed for three of the five NHPs. Two of the five NHPs self-resolved their infections without requiring clinical interventions.
- TP3 - Post-Peak: Observations and data collection 7 days after peak infection.
- TP4, TP6 and/or TP7 - Relapse: Sample collections to test expectations of a mild drop in haemoglobin levels and low parasitaemias relative to peak infection arising from activation of hypnozoites. Depending on the clinical outcomes of each subject, samples collected for relapses were classified as TPs 4, 6, and/or 7 (Table 1).
- TP4, TP5, TP6 and/or TP7 - Inter-Relapse: Inter-relapse interval. Depending on the clinical outcomes of each subject, samples collected for inter-relapses were classified as TPs 4, 5, 6, and/or 7 (Table 1).
- TP7 – Final: Sample collection immediately prior to the administration of radical curative treatment (primaquine + chloroquine) was always referred to as ‘TP7’. Depending on the clinical outcomes of each subject, TP7 may be classified as either relapse or inter-relapse.

**Table 1:** E04 Clinically assessed disease progression per time point for each subject.

	Baseline (Pre-infection)	Acute infection	Post-peak	Inter-relapse	Relapse
RFa14	TP1	TP2	TP3	TP4, TP5, TP6	TP7
RMe14	TP1	TP2	TP3	TP4, TP5, TP7	TP6
RIc14	TP1	TP2	TP3	TP5, TP7	TP4, TP6
RSb14	TP1	TP2	TP3	TP5, TP7	TP4, TP6
RFv13	TP1	TP2	TP3	N/A	N/A

E04 Data types and sample counts

*E04 Clinical, haematological & parasitological sample counts (428 total)*

Timepoint WB and daily WC were used for several clinical analyses. CBCs, reticulocyte counts, and blood smear slides to determine parasitaemias were generated. Blood chemistries were also performed. The following samples were analysed:

- 4 NHPs * 101 days * 1 Specimen type either (WB) or (WC) = 404 (includes 1 extra day before infection)
- 1 NHP * 24 days * 1 Specimen type either (WB) or (WC) = 24 (includes 1 extra day before infection; animal euthanised on day 23 post-infection)

*E04 Immune profiling sample counts (412 total)*

Samples from the 7 major TPs as well as WC and PC samples collected on select days were used for innate immune profiling with panels X, Y, Z. Adaptive immune profiling used panels A, B, C, D, and E (see methods). The following samples were analysed:

- 4 NHPs * 7 TPs * 3 panels (X, Y, Z) * 2 specimen types (WB, PL) = 168
- 1 NHP * 3 TPs * 3 panels (X, Y, Z) * 2 specimen types (WB, PL) = 18 (animal was euthanised)
- 5 NHPs * 4 days * 1 panel (Z) * 2 specimen types (WC, PC) = 40
- 4 NHPs * 7 TPs * 4 panels (A, B, C, D) * 1 specimen type (WB) = 112
- 4 NHPs * 7 TPs * 1 panel (E) * 1 specimen type (BM) = 28
- 1 NHP * 3 TPs * 4 panels (A, B, C, D) * 1 specimen type (WB) = 12 (animal was euthanised)
- 1 NHP * 3 TPs * 1 panel (E) * 1 specimen type (BM) = 3 (animal was euthanised)

Concentrations of 45 select cytokines were measured (see detailed methods). ELISA-determined concentrations of erythropoietin (EPO) were also measured. The following venous blood samples were analysed:

- 4 NHPs * 7 TPs * 1 specimen type (PS) = 28
- 1 NHP * 3 TPs * 1 specimen type (PS) = 3 (animal was euthanised)

*E04 Transcriptomics sample counts (63 total)*

The following samples were analysed:

- 4 NHPs * 7 TPs * 2 specimen types (WB, BM) = 56
- 1 NHP * 3 TPs * 2 specimen types (WB, BM) = 6 (animal was euthanised)
- 1 WB sample from a transfusion donor *M. mulatta* NHP CF97 = 1

*E04R Transcriptomics sample counts (33 total)*

E04R reflects resequencing of the RNA from the same (WB) samples (not BM) from E04 plus a sample from a control *M. mulatta* NHP, REe6. E04R resequencing used SOPs and technology consistent with E23R, E24, and E25 to make the results comparable. The following samples were analysed:

- 4 NHPs * 7 TPs * 1 specimen type (WB) = 28
- 1 NHP * 3 TPs * 1 specimen type (WB) = 3 (animal was euthanised)
- 1 WB sample from the transfusion donor NHP CF97 = 1
- 1 WB sample from the uninfected control NHP REe6 = 1

*E04 Metabolomics sample counts (260 total)*

The following capillary blood samples were analysed:

- 4 NHPs * 7 TPs * 1 specimen type (PS) = 28
- 1 NHP * 3 TPs * 1 specimen type (PS) = 3 (animal was euthanised, see E04 clinical section above)
- 4 NHPs * 48 every other day * 1 specimen type (PS) = 192
- 1 NHP * 10 every other day * 1 specimen type (PS) = 10 (animal was euthanised)
- 1 extra sample for RMe14 (PS) = 1
- 1 extra sample for RFa14 (PS) = 1
- 1 sample from the transfusion donor (PS) = 1
- 1 reference sample at the beginning and end of each batch (PS) = 2 * 12 = 24

*E04 Lipidomics sample counts (62 total)*

Multiple targeted lipid classes (see methods) were quantified. The following samples were analysed:

- 4 NHPs * 7 TPs * 2 specimen types (WB, BM) = 56
- 1 NHP * 3 TPs * 2 specimen types (WB, BM) = 6 (animal was euthanised)

*E04 Proteomics sample counts (31 total)*

The following red blood cell membrane (MN) samples were analysed:

- 4 NHPs * 7 TPs * 1 specimen type (MN) = 28
- 1 NHP * 3 TPs * 1 specimen type (MN) = 3 (animal was euthanised)

Note that results contain additional samples from E18, an uninfected control (not included in this paper).

- 1 NHP * 7 TPs * 1 specimen type (MN) = 7 from E18

### Experiment 23 (E23): *M. mulatta* infections with *P. cynomolgi* M/B strain to produce and integrate clinical, haematological, parasitological, and omics measures of acute primary infection and relapses

#### E23 Experimental Description

Malaria-naive *M. mulatta* (n = 6) (IDs: RBg14, ROc14, RJn13, RIb13, RAd14, ROh14), approximately four years of age, were assigned to this experiment, which was conducted from December 17^th^, 2014 to July 8^th^, 2015. On January 27^th^, 2015, after baseline blood and BM sampling and testing, these NHPs were inoculated intravenously with 2000 *P. cynomolgi* M/B strain salivary gland sporozoites produced and isolated at the CDC from multiple *Anopheles* species (*An. dirus, An. gambiae*, and *An. stephensi*) and then profiled for clinical, haematological, parasitological, immunological, transcriptomic, metabolomic, lipidomic, and proteomic measurements^18^. The experiment was designed for about 100 days, with pre- and post-100-day periods to prepare subjects and administer curative treatments, respectively. During the 100-day experimental period, subjects experienced days of patent and sub-patent infection. The anti-malarial drug artemether was subcuratively administered to subjects after the initial peak of infection if subjects were not able to self-resolve their infection. Blood-stage curative artemether was administered to all subjects following peak infection and following a period of relapse infection. All peaks were clinically determined for each subject. The anti-malarial drugs primaquine and chloroquine were administered to all subjects at the end of the study for curative treatment of the liver and blood-stage infections, respectively. Capillary blood samples were collected daily for the measurement of CBCs, reticulocytes, and parasitaemias. Additional capillary blood sample volume was collected every other day to obtain PS for metabolomic analysis. WB and BM samples were collected at seven TPs for transcriptomic, proteomic, lipidomic, and immunological analyses.

Experiment 23 is an iteration of E04, with the same parasite-host combination, but with some sampling and treatment adjustments. E23 is the first in a series of three experiments that includes subsequent homologous (E24, *P. cynomolgi* M/B strain sporozoites) and heterologous (E25, *P. cynomolgi* Ceylon strain sporozoites) challenges of the same individuals as in the E23 cohort, in each case with an inoculum of 2,000 sporozoites^1^. One of the six subjects from E23 was not included in subsequent experiments (E24 and E25) due to persistent behavioral issues that prevented effective quality sample collections. Transcriptomics results from E23 are also referred to as E23R, representing resequencing of the RNA samples from E23, processed with SOPs and technology consistent with those used for E04R, E24 and E25 so that results from these experiments are comparable. Unlike the E04 and E04R transcriptomics results, only E23R transcriptomics data (and not the original E23 transcriptomics data) have been deposited in the PlasmoDB database and used in analyses published to date^18^.

**Fig. 4:**
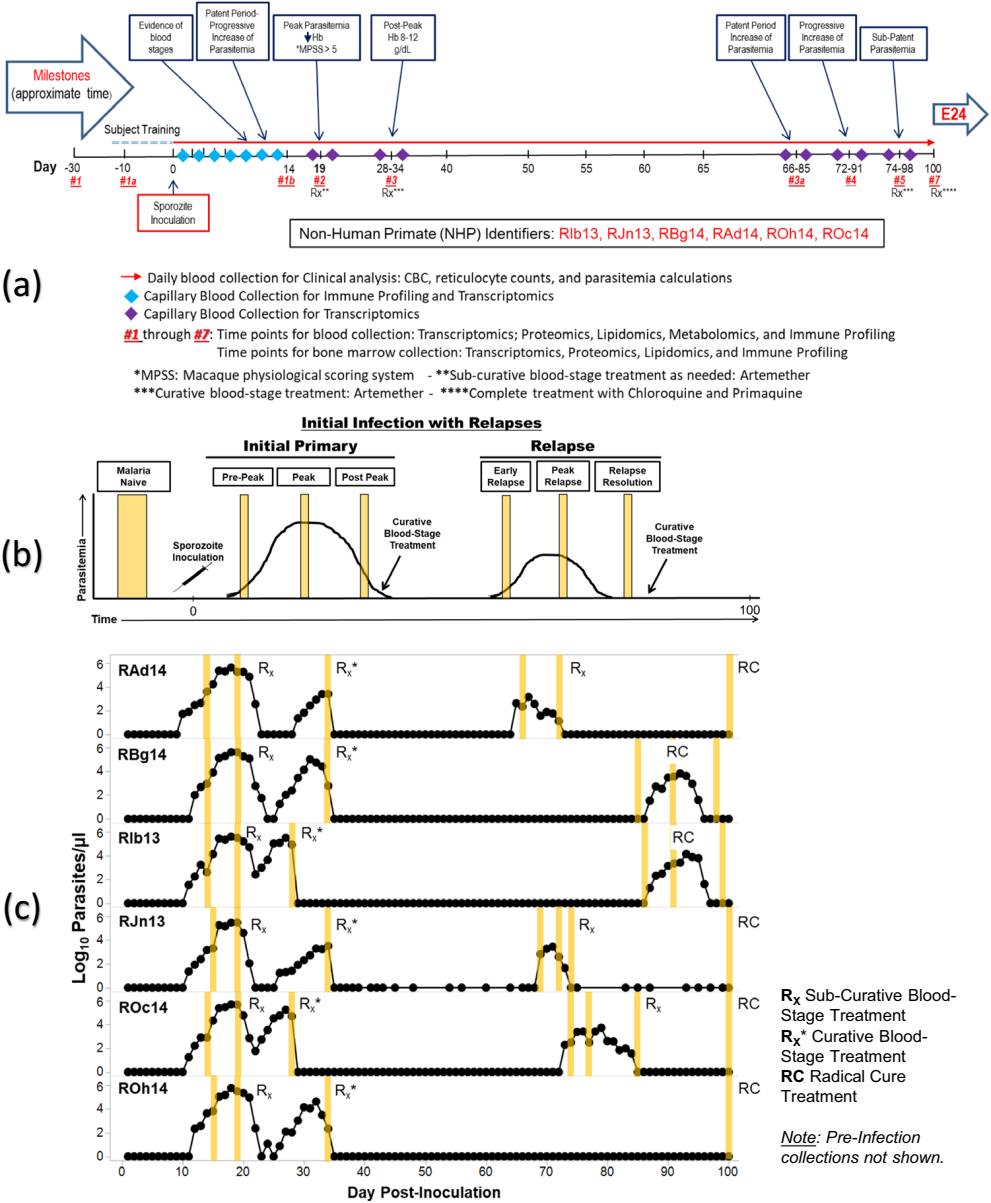
E23 projected ‘idealized’ and ‘actual’ experimental timeline and TP sample collection. (**a**) Timeline used to plan the experiment; (**b**) Figure showing ‘Idealized’ clinical progression for the infected NHP cohort, and (**c**) Figure showing ‘actual’ clinical progression that occurred during the experiment for each NHP in the cohort. The vertical yellow lines indicate actual timepoints as in Table 2. b and c are reproduced^18^ with changes and permission from the authors under the Creative Commons license CC BY 4.0.

Experiment 23 sample collection TPs and a representation of both the Idealized and Actual clinical progression of infection are included in Fig. 4, Table 2, and in the supporting clinical information. A summary of samples per NHP is available in Supplementary Table 3.

**Table 2:** E23 Clinically assessed disease progression per time point for each subject.

	Malaria naïve 1 (pre-infection)	Malaria naïve 2 (pre-infection)	Pre-peak	Peak	Post-peak	Early relapse	Peak relapse	Relapse resolution	Final
RAd14	TP1	TP1A	TP1B	TP2	TP3	TP3A	N/A	TP5	TP7
RBg14	TP1	TP1A	TP1B	TP2	TP3	TP3A	TP4	TP5	N/A
RIb13	TP1	TP1A	TP1B	TP2	TP3	TP3A	TP4	TP5	N/A
RJn13	TP1	TP1A	TP1B	TP2	TP3	TP3A	TP4	TP5	TP7
ROc14	TP1	TP1A	TP1B	TP2	TP3	TP3A	TP4	TP5	TP7
ROh14	TP1	TP1A	TP1B	TP2	TP3	N/A	N/A	N/A	TP7

Briefly, the TPs collected are defined as:

- TP1 - Malaria Naïve 1 (Pre-infection) - Uninfected control for each NHP.
- TP1A - Malaria Naïve 1 (Pre-infection) - NHP environment acclimation and sample extraction training.
- TP1B - Pre-Peak -This time point examined NHP responses after sporozoite inoculation with the *P. cynomolgi* M/B strain prior to the peak of parasitaemia. Activation of cellular and immune responses relative to baseline readings expected.
- TP2 - Peak - Peak of infection determined by clinical, haematological and parasitological assessment for each NHP. The time point characterized host immune response and captured omics measurements during the peak of parasitaemias when NHPs experienced clinical signs of disease, commonly severe in *P. cynomolgi* infections. NHPs were monitored based on clinical and haematological parameters, and clinical interventions with sub-curative doses of artemether and fluid support were performed as necessary.
- TP3 - Post-Peak - Observations seven days after peak of infection and sub-curative treatment. Haemoglobin levels monitored for decreases that could indicate decreased red blood cell (RBC) production, or abnormal destruction of uninfected RBCs.
- TP3A - Early Relapse - Observations of early relapse.
- TP4 - Peak Relapse - Observations of peak relapse.
- TP5 - Relapse Resolution - Low levels of persistent parasitaemia that may be below the microscopic detection threshold.
- TP6 - N/A (Planned second relapse not executed) - NHPs did not experience a second relapse, and no samples were collected.
- TP7 - Final - Low levels of persistent parasitaemia that may be below the microscopic detection threshold. Samples are representative of the period between relapses before curative treatment with chloroquine and primaquine.

E23 Data types and sample counts

*E23 Clinical, haematological & parasitological sample counts (590 total)*

Daily WC were used for several clinical analyses. CBCs, reticulocyte counts, and blood smear slides to determine parasitaemias were generated. Blood chemistries were also performed on TPs and select days. The following samples were analysed:

- 5 NHPs * 102 days * 1 specimen type (WC) = 510 (including 2 baseline days before infection)
- 1 NHP * 80 days * 1 specimen type (WC) = 80 (including 2 baseline days before infection)

*E23 Immune profiling sample counts (1186 total)*

Three flow cytometry panels (X, Y, Z) designed to study the innate immune responses. Peripheral blood mononuclear cells (PB) derived from WB collected at major TPs were used for adaptive immune profiling with 7 flow cytometry panels (C, D, I, J, M, N, L) The following samples were analysed:

- 6 NHPs * 3 panels (X, Y, Z) * 2 specimen types (PC, WC) * 7 select days = 252
- 6 NHPs * 3 panels (X, Y, Z) * 4 specimen types (PC, PL, WC, WB) * 9 TPs (unequally collected overall all NHPs, see usage notes for details) = 571
- 6 NHPs * 7 panels (C, D, I, J, M, N, L) * 1 specimen type (PB) * 9TPs (unequally collected overall all NHPs, see usage notes for details) = 273

Concentrations of 45 select cytokines were measured using the venous PS collected at up to 9 TPs. A total of 42 samples were analysed.

Venous PS collected at 9 major TPs were used to measure the concentrations of IgG and IgM antibodies specific for *P. cynomolgi* infected red blood cells and uninfected red blood cells as determined by ELISA in addition to the total plasma concentration of IgG and IgM. A total of 48 samples were analysed.

*E23R Transcriptomics sample counts (48 total)*

The following samples were analysed:

- 6 NHPs * 9 TPs (unequally collected overall all NHPs, see usage notes for details) * 1 specimen type (WB) = 48

*E23 Metabolomics sample counts (388 total)*

Reference NHP samples, metabolomics standards and the following capillary blood samples were analysed:

- 6 NHPs * 1 specimen type (PS) * 6 TPs (unequally collected overall all NHPs, see usage notes for details) = 29
- 6 NHPs * 1 specimen type (PS) * 9 TPs (unequally collected overall all NHPs, see usage notes for details) = 48
- 6 NHPs * 1 specimen type (PS) * every other day (unequally collected overall all NHPs, see usage notes for details) = 259
- Reference (PS) & Standards (PS) = 52

*E23 Lipidomics sample counts (94 total)*

Multiple targeted lipid classes (see methods) were quantified. The following samples were analysed:

- 6 NHPs * 1 specimen type (WB) * 9 TPs (unequally collected overall all NHPs, see usage notes for details) = 48
- 6 NHPs * 1 specimen type (BM) * 9 TPs (unequally collected overall all NHPs, see usage notes for details) = 46

*E23 Proteomics sample counts (24 total)*

Analyses were performed on the first 4 TPs (TP1, TP1A, TP1B, TP2). Note that proteomics data were not generated for further time points (TPs 3–7).

The following samples were analysed:

- 6 NHPs * 4 TPs * 1 specimen type (MN) = 24

### Experiment 24 (E24). *M. mulatta* infected with *P. cynomolgi* M/B strain, in a homologous challenge, to produce and integrate clinical, haematological, parasitological, immune response and omics measures from acute primary infection and relapses

#### E24 Experimental Description (homologous strain reinfection experiment)

*Macaca mulatta* (n = 5) (IDs: RBg14, ROc14, RIb13, RAd14, ROh14), approximately five years of age and cleared of their previous E23 infection with *P. cynomolgi* M/B strain via treatment with the anti-malarial drugs artemether, chloroquine, and primaquine, were inoculated on July 21^st^, 2015, (2 months after curative treatments following E23^1^) again intravenously with 2,000 *P. cynomolgi* M/B strain salivary gland sporozoites produced and isolated at the CDC from multiple *Anopheles* species (*An. dirus*, *An. gambiae*, and *An. stephensi*) and then profiled for clinical, haematological, parasitological, immunological, and transcriptomic measurements. They were studied from July 20^th^, 2015 to September 3^rd^, 2015. E24 included 1 pre-inoculation day, 35 experiment days, and 10 post-experiment days^18^. The anti-malarial drugs primaquine and chloroquine were administered to all subjects at the end of the study for curative treatment of the liver and blood-stage infections, respectively. Capillary blood samples were collected daily for the measurement of CBCs, reticulocytes, and parasitaemias. Capillary blood samples were collected every other day to obtain capillary PS for metabolomic analysis. Venous blood samples were collected at three TPs for immunological, transcriptomic, and lipidomic analyses. This is the second in a series of experiments that includes infection of malaria-naive subjects with *P. cynomolgi* M/B strain (E23), followed by infection of the same animals with the same strain (E24), and then a heterologous strain challenge (E25, *P. cynomolgi* Ceylon strain).

**Fig. 5:**
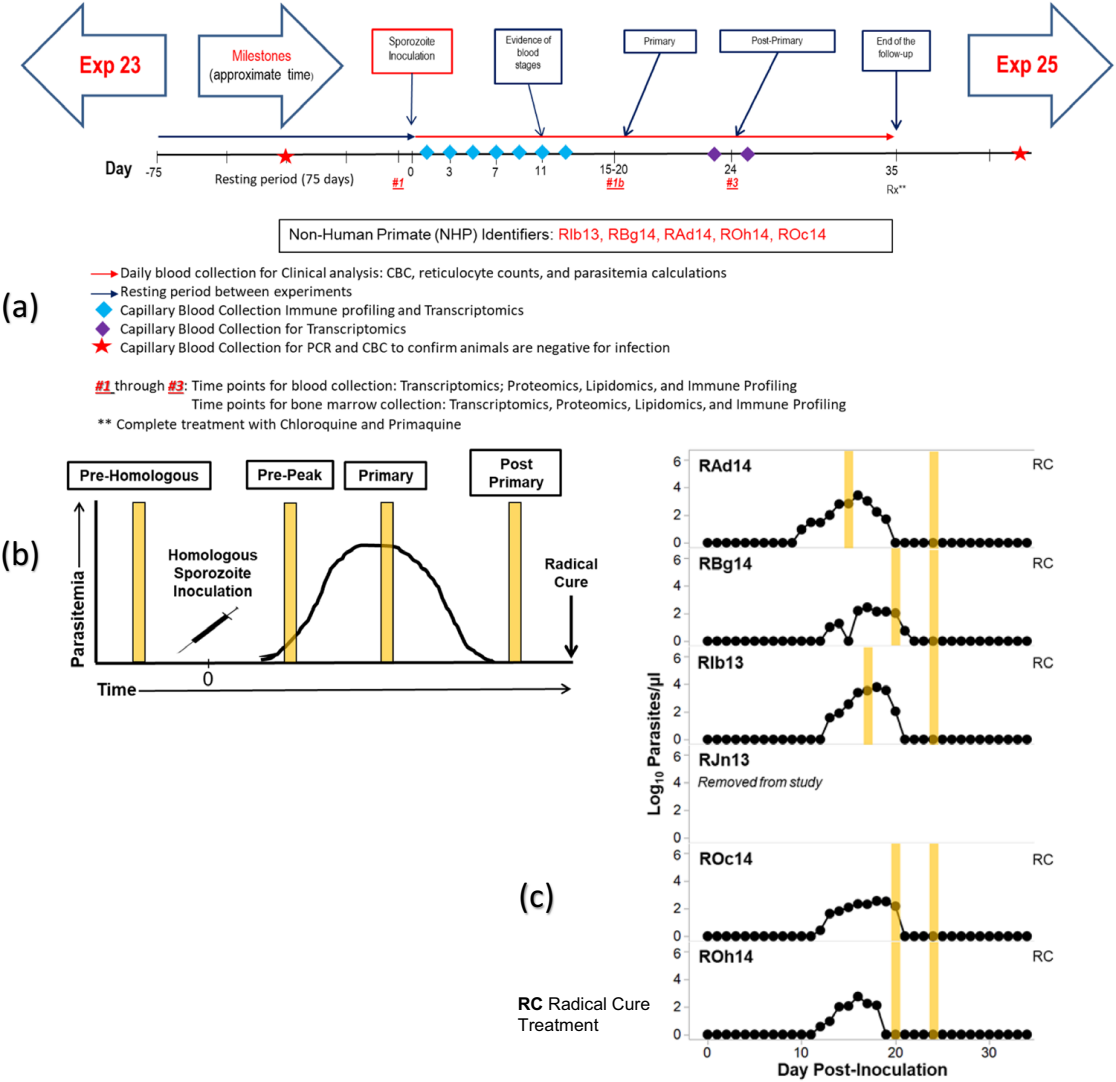
E24 projected ‘idealized’ and ‘actual’ experimental timeline and TP sample collection. (**a**) Timeline used to plan the experiment; (**b**) Figure showing Idealized clinical progression for the infected NHP cohort, and (**c**) Figure showing actual clinical progression that occurred during the experiment for each NHP in the cohort. The vertical yellow lines indicate actual timepoints as in Table 3. b and c are reproduced^18^ with changes and permission from the authors under the Creative Commons license CC BY 4.0.

Sample collection TPs and a representation of both the Idealized and Actual clinical progression of infection are included in Fig. 5, Table 3, and in the supporting clinical information. A summary of samples per NHP is available in Supplementary Table 4.

**Table 3:** E24 Clinically assessed disease progression per time point for each subject.

	Pre-homologous (Pre-infection)	Pre-peak	Primary	Post-primary
RAd14	TP1	N/A	TP1B	TP3
RBg14	TP1	N/A	TP1B	TP3
RIb13	TP1	N/A	TP1B	TP3
ROc14	TP1	N/A	TP1B	TP3
ROh14	TP1	N/A	TP1B	TP3

Briefly, the TPs collected are defined as:

- TP1 - Pre-Homologous (Pre-Infection) - Pre-infection stage of an NHP completely treated to resolve blood and liver stage infections. The appropriate baseline naïve control to this time point will be Experiment 23, TPs 1 and 1A.
- TP1A - Pre-Peak - N/A (planned but not observed/collected)
- TP1B - Primary - Examined responses after sporozoite re-inoculation with the homologous (relative to E23) *P. cynomolgi* M/B strain prior to the peak of parasitaemia. Activation of cellular and immune responses relative to baseline readings expected.
- TP3 - Post-Primary - Planned to examine expected non-severe host immune and metabolic responses during the peak of infection and clinical signs of disease. NHPs were monitored based on clinical and haematological parameters, and clinical interventions were performed as necessary if parasitaemias were not self-resolved. Note: This was planned to be a peak parasitaemia collection TP, but animals self-controlled infection more quickly than expected. Therefore, the days with the highest parasite densities were missed, and parasitaemias were sub-microscopic in all animals. Time point names are updated to reflect this.

E24 Data Types and sample counts

*E24 Clinical, haematological & parasitological sample counts (195 total)*

Daily WC were used for several clinical analyses. CBCs, reticulocyte counts, and blood smear slides to determine parasitaemias were generated. Blood chemistries were also performed on TP days. The following samples were analysed:

- 5 NHPs * 36 days * 1 specimen type (WC) = 180
- 5 NHPs * 3 TPs * 1 blood chemistry (WB) = 15

*E24 Immune profiling sample counts (525 total)*

Three flow cytometry panels (X, Y, Z) designed to study the innate immune responses. WB and PB derived from WB collected at major TPs were used for adaptive immune profiling with 7 flow cytometry panels 7 flow cytometry panels (C, D, AD, AE, AA, N, S). The following samples were analysed:

- 5 NHPs * 3 panels (X, Y, Z) * 4 specimen types (PC, PL, WC, WB) * 3 TPs = 180
- 5 NHPs * 3 panels (X, Y, Z) * 2 specimen types (PC, WC) * 7 days = 210
- 5 NHPs * 4 panels (D, C, AD, S) * 1 specimen type (WB) * 3 TPs = 60
- 5 NHPs * 3 panels (AE, AA, N) * 1 specimen type (PB) * 3 TPs = 45

Concentrations of 45 selected cytokines were measured using the venous PS samples collected at 3 TPs. A total of 15 samples were analysed.

Venous PS samples collected at 3 TPs were used to measure the concentrations of IgG and IgM antibodies specific for *P. cynomolgi* infected RBCs and uninfected RBCs as determined by ELISA in addition to the total plasma concentration of IgG and IgM. A total of 15 samples were analysed.

*E24 Transcriptomics sample counts (15 total)*

The following samples were analysed:

- 5 NHPs * 3 TPs * 1 sample type (WB) = 15

### Experiment 25 (E25). *M. mulatta* infected with *P. cynomolgi* Ceylon strain, in a heterologous challenge, to produce and integrate clinical, haematological, parasitological, immunological, and omics measures of acute primary infection and relapses

#### E25 Experimental description: (heterologous reinfection experiment)

*Macaca mulatta* from E23/E24 (n = 5), approximately five years of age and cleared of previous E24 infection with *P. cynomolgi* M/B strain via treatment with the anti-malarial drugs artemether, chloroquine, and primaquine, were inoculated – about 60 days later – intravenously with 2,000 salivary gland sporozoites produced and isolated at the CDC from multiple *Anopheles* species (*An. dirus*, *An. gambiae*, and *An. stephensi*) and then profiled for clinical, haematological, parasitological, immunological, transcriptomic, lipidomic, and proteomic measurements^1^. The experiment included 8 pre-inoculation days, 49 experiment days, and 4 post-experiment days. The anti-malarial drug artemether was subcuratively administered to subjects at the initial peak of infection if subjects were not able to self-resolve their parasitaemias. Peak infection was determined clinically for each subject. The anti-malarial drugs primaquine and chloroquine were administered to all subjects at the end of the study for curative treatment of the liver and blood-stage infections, respectively. Capillary blood samples were collected daily for the measurement of CBCs, reticulocytes, and parasitaemias. Capillary blood samples were collected every other day to obtain PS for metabolomic analysis. Venous blood samples were collected at five TPs for immunological, transcriptomic, lipidomic, and proteomic analyses. Experiment 25 is the last in a series of experiments that had first included infection of malaria-naive subjects (Experiment 23, *P. cynomolgi* M/B strain) and homologous challenge (Experiment 24, *P. cynomolgi* M/B strain) of individuals from the same cohort.

**Fig. 6:**
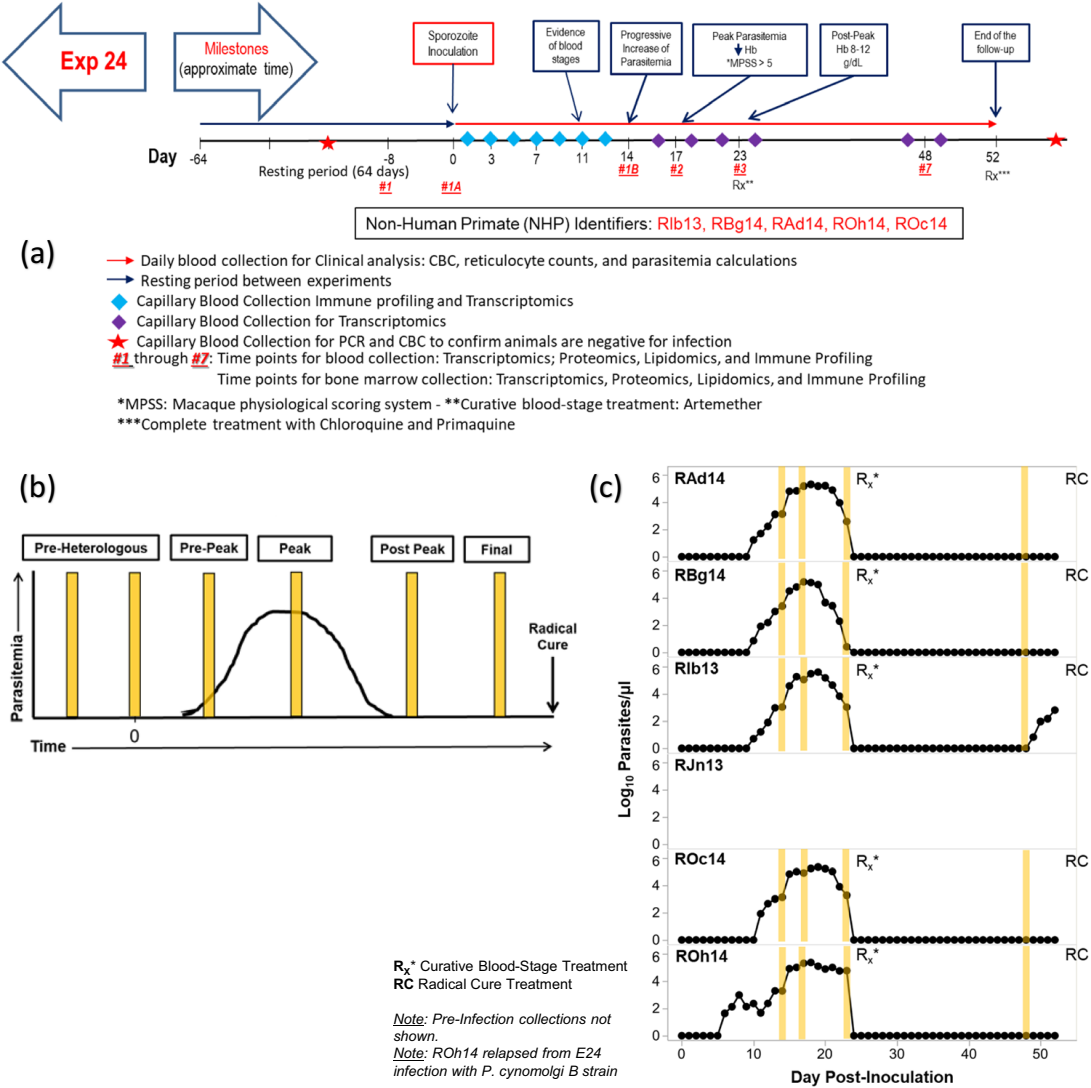
E25 planned and actual experimental timeline and TP sample collection. (**a**) Timeline used to plan the experiment; (**b**) ‘Idealized’ figure showing projected clinical progression for the infected NHP cohort, and (**c**) ‘Actual’ figure showing clinical progression that occurred during the experiment for each NHP in the cohort. The vertical yellow lines indicate actual timepoints as in Table 4.

E25 involved five NHP subjects (IDs: RBg14, ROc14, RIb13, RAd14, ROh14), which were studied from October 19^th^, 2015 to December 18^th^, 2015. The NHPs were infected on October 27^th^, 2015. Sample collection TPs and a representation of both the Idealized and Actual clinical progression of infection are included in Fig. 6, Table 4, and in the supporting clinical information. A summary of samples per NHP is available in Supplementary Table 5.

**Table 4:** E25 Clinically assessed disease progression per time point for each subject.

	Pre-heterologous (Pre-infection)	Pre-heterologous (Pre-infection)	Pre-peak	Peak	Post-peak	Final
RAd14	TP1	TP1A	TP1B	TP2	TP3	TP7
RBg14	TP1	TP1A	TP1B	TP2	TP3	TP7
RIb13	TP1	TP1A	TP1B	TP2	TP3	TP7
ROc14	TP1	TP1A	TP1B	TP2	TP3	TP7
ROh14	TP1	TP1A	TP1B	TP2	TP3	TP7

Briefly, the TPs collected are defined as:

- TP1 - Pre-Heterologous-1 (Pre-Infection) - Pre-infection baseline sample collection from NHPs after treatment to resolve prior E24 blood- and liver-stage infections.
- TP1A - Pre-Heterologous-2 (Pre-Infection) - Second pre-infection baseline collection on the same day as sporozoite inoculation. Note that the TP1 immunology sample was not analysed due to a sample handling error. For this reason, TP1A was collected exclusively for immunology analysis, and only the immunology dataset contains results from TP1A.
- TP1B - Pre-peak – This TP examined NHP responses after sporozoite inoculation with the heterologous Ceylon strain prior to the peak of parasitaemia, expecting to see activation at a cellular and immune level compared to baseline readings.
- TP2 - Peak - This TP examined host immune and metabolic response during the peak of infection and clinical signs of disease. The response was expected to be severe or non-severe depending on the subject. The NHPs were monitored based on clinical and haematological parameters, and clinical interventions were performed as necessary if hyperparasitaemia did not self-resolve.
- TP3 - Post-Peak - Observations seven days after the peak of infection and curative blood-stage treatment if needed.
- TP4-6 - N/A – These TPs did not apply, as the NHPs did not relapse after sub-curative blood-stage treatment.
- TP7 - Final - Determined clinical status of NHPs at the end of the experiment to complete anti-malaria treatment.

E25 Data types and sample counts

*E25 Clinical, haematological & parasitological sample counts (335 total)*

Daily WC were used for several clinical analyses. CBCs, reticulocyte counts, and blood smear slides to determine parasitaemias were generated. Blood chemistries were also performed on TP days. The following samples were analysed:

- 5 NHPs * 59 days * 1 specimen type (WC) = 295
- 5 NHPs * (3 TP + 5 daily collections) for blood chemistry = 40

*E25 Immune profiling sample counts (720 total)*

Three flow cytometry panels (X, Y, Z) designed to study the innate immune responses. WB and PB derived from WB collected at major TPs were used for adaptive immune profiling with 7 flow cytometry panels 7 flow cytometry panels (C, D, AD, AE, AA, N, S). The following samples were analysed:

- 5 NHPs * 3 panels (X, Y, Z) * 4 specimen types (PC, PL, WC, WB) * 5 TPs = 300
- 5 NHPs * 3 panels (X, Y, Z) *1 specimen type (WC) * 7 days = 105
- 5 NHPs * 3 panels (X, Y, Z) *1 specimen type (PC) * 6 days = 90
- 5 NHPs * 2 panels (D, C) * 1 specimen type (WB) * 5 TPs = 50
- 5 NHPs * 5 panels (AD, AE, AA, N, S) * 1 specimen type (PB) * 5 TPs = 125

Concentrations of 45 selected cytokines were measured using the venous PS samples collected at 5 major TPs. A total of 25 samples were analyzed.

Venous PS collected at 3 TPs were used to measure the concentrations of IgG and IgM antibodies specific for *P. cynomolgi* infected RBCs and uninfected RBCs as determined by ELISA, in addition to the total plasma concentration of IgG and IgM. A total of 25 samples were analyzed.

*E25 Transcriptomics sample counts (25 total)*

The following samples were analysed:

- 5 NHPs * 5 TPs * 1 specimen type (WB) = 25

*E25 Lipidomics sample counts (25 total)*

Multiple targeted lipid classes (see methods) were quantified. The following samples were analysed:

- 5 NHPs * 5 TPs * 1 specimen type (WB) = 25

### Detailed Experimental methods and data analyses for E13, E04, E04R, E23R, E24 and, E25

Clinical, haematological and parasitological (CBCs, chemistry profiles, cell counts via microscopy and haematology analyser)

Blood samples were analysed for cell counts and calculations of CBCs, normoblasts, reticulocytes, and parasitaemias. In addition to these results, veterinary data and metadata were collected on all facets of animal access, including, but not limited to treatments, haematology, biochemical analyses, parasitology, bacteriology, and surgery statistics, etc. These veterinary data types were collected from the time of animal assignment to the MaHPIC at the YNPRC to the end of specific experiments and unassignment of animals, after any final curative treatments. SOPs including analytical metadata (e.g., instrument and technician information, reagent lot numbers, etc.) are available for haematology analysis; blood smears and parasitaemia calculations; and normoblast count corrections. The veterinary datasets also contain files that describe any recorded daily activities, instances of cage washing, and any noteworthy observations about the NHPs during sample collections (e.g., any unusual physical or behavior issues). Note E13 blood samples were only analysed for CBC and reticulocyte counts.

#### CBCs, normoblast, parasitaemia, iSTAT and reticulocytes

With some deviations depending on the experiment, data were collected as follows: haematology parameters as observed by a CBC using a haematology analyser, chemistry profiles using an iSTAT and chemistry analysers, parasite counts obtained from reading thin and thick blood smear slides, reticulocyte counts obtained from manual readings, BM cell counts using a haematology analyser, and coagulation assays.

CBCs were calculated on a Beckman Coulter AcTDiff analyser (serial number 6706366). Quality control was performed at three control levels: abnormal low, normal, and abnormal high (control catalog number 7547188) with three runs for each control type (lot numbers included with dataset analytical metadata). When normoblasts (maturing nucleated RBCs) were present in the peripheral blood, they were identified by the CBC instrument as monocytes or lymphocytes. Thin blood smear readings were used as a backup method to determine actual normoblast counts (see below).

Parasitaemias were calculated as the number of parasites per microliter. Infected RBC counts were taken as the average counts of two readers derived from reading either a thick or thin blood smear. For cases where there was significant variation between the readers, a third reader was employed and the average of two closest readings were recorded as the final count. Blood smears were created with 5 μl of blood for thin smears, and 3 μl of blood for thick smears, using WB mixed with EDTA. Thin smears were fixed with ethanol. The smears were stained with Giemsa stain. The infected RBCs were counted by microscopy using oil immersion and World Health Organization Malaria Microscopy protocols^19^. The dataset contains the specific SOP for generating the counts. Parasite counts were normalized as follows:

$${\rm{Parasites}}/{\rm{\mu l}}=\frac{{\rm{Total}}\;{\rm{number}}\;{\rm{RBCs}}\;\,({\rm{from}}\;{\rm{CBC}})\,\times {\rm{infected}}\;{\rm{RBCs}}\;\,({\rm{from}}\;{\rm{thin}}\;{\rm{blood}}\;{\rm{smear}}\;{\rm{counts}})\,}{{\rm{RBCs}}\;{\rm{counted}}\;{\rm{from}}\;{\rm{thin}}\;{\rm{blood}}\;{\rm{smear}}}$$

The purpose of these infections was to study relapsing infections caused by the activation of hypnozoites in the liver that cause a subsequent blood-stage parasitemia. This is in contrast to a recrudescence where the parasites persist in the blood and fluctuate above and below the level of detection. While PCR is advantageous to distinguish these two infection types, it was not performed here because the level of detection for PCR and microscopy were comparable for the assays that were available. Nevertheless, our infections are still considered to be relapses and not recrudescences because they exhibit similar patterns as prior published work with the *P. cynomolgi* M/B strain^20^.

Normoblasts are nucleated immature RBCs in the peripheral blood. The dataset contains the specific SOP for normoblast counts. When normoblasts are present in the peripheral blood, they are identified by haematology analysers as monocytes or lymphocytes. Thin blood smears were therefore scanned for the presence of immature RBCs. If they were detected, differential cell counts were performed, and WBC counts were corrected to adjust for the normoblast counts. The SOP for this correction is included in the dataset. Briefly, if a single normoblast was observed on the thin blood smear preparation, the total number of WBCs was corrected as follows

$${\rm{Corrected}}\;{\rm{WBCs}}\;{\rm{Count}}=\frac{{\rm{Absolute}}\;\#\;{\rm{WBC}}\;({\rm{from}}\;{\rm{CBC}})}{(\#\;{\rm{Normoblast}}\;{\rm{count}}/100)+1}$$

Reticulocytes are immature RBCs, comprising about 1% of the R BCs in humans. Reticulocytes develop and mature in the BM and then circulate for about a day in the blood before becoming mature RBCs. The reticulocyte count rises when there is significant blood loss or in certain diseases like malaria in which RBCs are destroyed prematurely. Reticulocytes numbers were calculated from thin blood smears. The dataset contains the specific SOP for reticulocyte counts.

#### iSTAT Blood Chemistry Protocol

Venous and capillary samples were collected at point of care during acute infection for biochemistry testing of electrolytes, chemistries, blood gases, and hematological analytes. Whole blood was placed in a single-use disposable iSTAT Chem8 + cartridge and analyzed using the iSTAT system analyzer (Abbott). The following analytes were reported: Sodium, Potassium, Chloride, Ionized Calcium, Glucose, BUN, Creatinine, Hematocrit, Hemoglobin, Anion GAP, and Total Carbon Dioxide concentration. The results from iSTAT analyzer were transmitted to a dedicated computer for data management by using the iSTAT downloader module. Quality controls for the iSTAT chem8 + cartridges were performed by using the iSTAT controls (TriControls level1 and level 3) and for the Handheld iSTAT system the internal and external electronic simulator was tested for every use or as needed for regulatory compliance.

#### Immunology (flow cytometry: E04, E23, E24, E25)

The following flow cytometry panels were designed to study the innate immune responses for each of these experiments:

- Panel X (PC or PL): Platelet Activation Status after Bacterial Stimulation
- Panel X (WC or WB): Neutrophil and Monocyte Activation Status after Bacterial Stimulation
- Panel Y (PC or PL): Platelet Activation Status after PMA Stimulation
- Panel Y (WC or WB): Neutrophil and Monocyte Activation Status after Stimulation with PMA
- Panel Z (PC or PL): Platelet Activation Status
- Panel Z (WC or WB): Neutrophil and Monocyte Activation Status

Additionally, depending on the experiment (as shown), the following flow cytometry panels were designed to study the adaptive immune responses:

Bone marrow panel:

- Panel E (BM) (E04): Leukocyte and Reticulocyte Dynamics in the BM

Monocyte and dendritic cell panels:

- Panel C (PB or WB) (E04, E23, E24, E25): Monocyte and Dendritic Cell Subset Phenotyping and Co-stimulatory Ability
- Panel D (PB or WB) (E04, E23, E24, E25): Monocyte and Dendritic Cell Subset Phenotyping and Co-stimulatory Ability (Isotype Control for Co-stimulatory Molecules)

Lymphocyte panels:

- Panel A (WB) (E04): Identification and Phenotyping of Lymphocyte Subsets
- Panel B (WB) (E04): Activation – Proliferation – Apoptosis of Lymphocytes

B cell panels:

- Panel I (PB) (E23): B cell phenotyping
- Panel J (PB) (E23): B cell intracellular
- Panel AD (PB or WB) (E24, E25): B cell immunoglobulin
- Panel AE (PB) (E24, E25): B cell proliferation and apoptosis

T cell panels:

- Panel M (PB) (E23): T cell phenotyping panel with chemokine receptor markers
- Panel N (PB) (E23, E24, E25): T cell phenotyping panel with intracellular staining for proliferation and apoptosis
- Panel L (PB) (E23): T cell phenotyping panel with chemokine receptor markers
- Panel AA (PB) (E24, E25): T cell phenotyping panel with chemokine receptor markers
- Panel S (PB or WB) (E24, E25): T cell trafficking panel, chemokines production

**Fig. 7:**
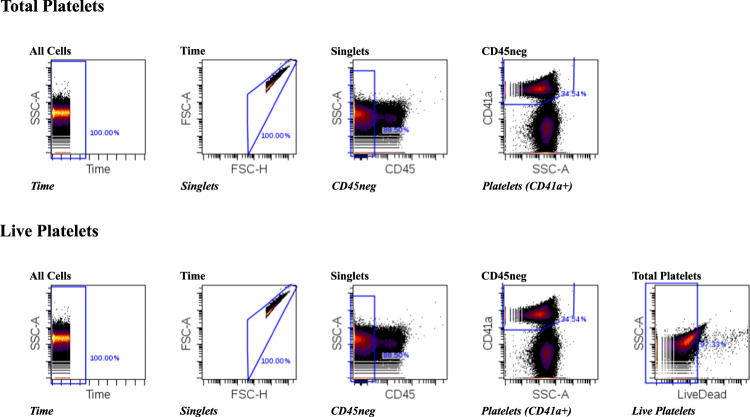
Flow cytometry gating strategy for E23 panel X, Y, Z platelets.

All flow cytometry assays were run on a BD LSR-II flow cytometer. Gating and data analyses were performed in Cytobank^21^. Gating strategies used for each panel are available in Immport as supplementary files for each experiment. As a reference, the gating strategy for panels X, Y, and Z in E23 is displayed in Fig. 7. Sample processing and staining procedures for individual panels are described below.

#### Staining procedure for panels X, Y, Z (E04, E23, E24, E25)

25 µl of capillary or WB each were used for panels Z, Y, and X. Molecular probes listed in Table 5 (approximately 5 μl master mix per tube) were added and left to incubate in 37 °C water bath for 30 min. Samples were removed from 37 °C water bath and placed on ice. Antibody mixtures listed in Table 6 (20 μl of master mix per tube) were added and incubated for 15 min on ice. The tubes were removed from ice, added an excess of phosphate buffered saline (PBS, approximately 2 ml), and spun at 400 × g for 10 min at 4 °C. The supernatant containing platelets was removed and placed in another pre-labeled FACS tube for platelets. The pellet was re-suspended in 2 ml of lyse-fix PhosFlow by pipetting up and down, followed by pulse vortexing voraciously. Tubes were then placed at 4 °C overnight or at least for 3 h to allow for lysing and fixing of the cells. After incubation, the tubes were centrifuged at 400 × g at 4 °C for 10 min, and the supernatant was aspirated and discarded. Pellet was resuspended in 200 µl of PBS. Tubes with platelet fraction were centrifuged at 3,000 × g for 10 min at 4 °C, and the supernatant was aspirated and discarded. The platelet pellet was resuspended in 200 µl of PBS and placed in 4 °C until needed for acquisition the next day.

**Table 5:** Molecular probes used in flow cytometry panels X, Y, and Z.

Probe	Fluorochrome	Clone	Remarks
CellROXDR	APC	N/A	
Live/Dead	Yellow	N/A	
pHrodo	FITC	N/A	Panel X only
FLICA	FITC	N/A	Panel Y, Z only

**Table 6:** Antibody master mixes used in flow cytometry panels X, Y, and Z.

Antibody	Fluorochrome	Clone	Remarks
CD16	PerCP-Cy5.5	3G8	
CD41	PE-Cy7	HIP8	
CD45	Alexa700	D058-1283	
CD63	Pacific Blue	H5C6	
CD64	APC-Cy7	10.1	E23, E24, E25 only
CD69	APC/Cy7	FN50	E04 only
CD66abce	PE	TET2	
Fc Block	N/A	N/A	

#### Staining procedure for panels A, B, C, D, E (E04)

200 µl WB or BM aspirate (panel E) were washed with 2 ml of sterile 1X PBS by centrifuging at 400 × g for 5 min at room temperature (RT), and the supernatant was aspirated and discarded. The remaining pellet was suspended in PBS 1x to match the original volume, and an appropriate amount of antibody master mix was added as indicated in Tables 7–10 for panels A, B, E, and C & D respectively, and incubated for 30 min in the dark at RT. 2 ml FACS Lysing solution was added and incubated for 10 min in the dark at RT. The remaining cells were then pelleted by centrifugation at 400 × g for 5 min, then washed twice in 2 mL of FACS buffer by centrifugation at 400 × g for 5 min. The supernatant was discarded, and cells were resuspended in a 2% paraformaldehyde and PBS solution (v/v).

**Table 7:** Flow cytometry panel A.

Antibody/probe	Fluorochrome	Clone	Remarks
CD3	Percp Cy5.5	SP-34-2	
CD4	FITC	L-200	
CD21	APC	B-LY4	
CD16	Alexa 700	3G8	
CD20	PE-Cy7	L27	
CD14	Pacific Blue (PB)	M5E2	
TCRgamma/delta	PE CF594	B1	
CD27	PE	M-T271	
CD8	V-500	SK1	
CD45	APC-CY7	D058-1283

**Table 8:** Flow cytometry panel B.

Antibody/probe	Fluorochrome	Clone	Remarks
CD3	Percp Cy5.5	SP-34-2	
CD4	FITC	L-200	
CD95	PE- CF594	DX2	
CD28	PE-CY7	CD-28.2	
PD1	PB	EH12.1	
CD8	V500 (Amcyan)	SK1	
CD20	Alexa 700	2H7	
Ki-67	APC = Alexa fluor 647	B56	Intracellular
Caspase 3	PE	C92-605	Intracellular
CD45	APC -Cy7	D058-1283

**Table 9:** Flow cytometry panel E.

Antibody/probe	Fluorochrome	Clone	Remarks
CD3	Percp Cy5.5	SP34-2	
CD45	FITC	DO58-1283	
CD41a	PE	HIP8	
CD71	APC	LOl.1	
CD11b	PE Cy7	ICRF44	
CD34	PE-TR (PE CF594)	563	
CD44	APC-H7 (CY7)	G44.26	
CD16	ALEXA 700	3G8	
CD14	PB	M5E2	
CD20	V-500 (Amcyan)	L27

**Table 10:** Flow cytometry panels C and D.

Antibody/probe	Fluorochrome	Clone	Remarks
CD11c	Alexa647	3.9	
CD123	PerCP-Cy5.5	7G3	
CD14	Pacific Blue	M5E2	
CD16	Alexa700	3G8	
CD20	FITC	L27	
CD3	FITC	SP34	
CD45	APC-Cy7	D058-1283	
CD8	FITC	SK-1	
CD80	PE-Cy7	L307.4	Panel C only
CD83	PE	HB15e	Panel C only
CD86	PECF594	Fun-1 (2331)	Panel C only
CD80 IgG1K	PE-Cy7	MOPC21	Panel D only
CD83 IgG1	PE	MOPC21	Panel D only
CD86 IgG1	PECF594	X40	Panel D only
HLA-DR	V500	G46-6	
Fc Block	N/A	N/A	E23, E24, E25 only
Live/Dead Fixable	Green	N/A	E23, E24, E25 only

For panel B, the following additional steps were performed for intracellular staining. 0.5 ml of BD-perm2 was added and incubated for 10 min in the dark at RT. The cells were then washed in 2 ml FACS buffer and centrifuged at 400 × g for 5 mins at RT. Antibodies for intracellular staining, as indicated in Table 4, were then added and incubated for 30 mins in the dark at RT. The cells were then washed in 2 ml of FACS buffer by centrifugation at 400 × g for 5 min. The supernatant was discarded, and cells were resuspended in 200 µl of PFA 1%.

#### Staining procedure for panels C, D (E24, E25), panels AA, S (E24)

200 µl WB were aliquoted for each panel into a 4 ml FACS Tube, 2 ml of sterile 1X PBS was added, was washed by centrifuging at 400 × g for 7 min at 4 °C, supernatant was aspirated and discarded. The pellet was resuspended in the appropriate amount of antibody master mix as indicated in the Tables 10–12 for panels C & D, AA, and S, respectively, and incubated on ice for 45 minutes. 2 ml of sterile 1X PBS was added and washed by centrifuging at 800 × g for 7 min at 4 °C, supernatant was aspirated and discarded. 2 ml of BD FACS Lysis buffer was added to each tube and pulse vortexed, incubated at RT for 10 minutes in the dark, centrifuged at 800 × g for 7 min at 4 °C, supernatant was aspirated and discarded. Cell pellet was resuspended in 1 ml of BD Lyse-Fix PhosFlow and stored at 4 °C overnight or for at least 1 h, tubes centrifuged at 800 × g for 7 min at 4 °C. All except 50 µl of supernatant was aspirated and discarded. Cells were resuspended in exactly 280 µl of PBS using the bubble method and 20 µl of Life Trucount Beads were added.

**Table 11:** Flow cytometry panel AA.

Antibody/probe	Fluorochrome	Clone	Remarks
BETA7	BV421	FIB504	
CCR7	FITC	150503	
CD14	BV605	M5E2	
CD20	BV605	2H7	
CD28	PE-Cy7	CD2-28.2	
CD3	PerCP-Cy5.5	SP-34-2	
CD4	PE	L-200	
CD45	APC-Cy7	D058-1283	
CD8	Alexa700	SK-1	
CD95	PECF594	DX2	
CXCR5	APC	MU5UBEE	
Fc Block	N/A	N/A	
Live/Dead Fixable	Yellow	N/A

**Table 12:** Flow cytometry panel S.

Antibody/probe	Fluorochrome	Clone	Remarks
CCR5	APC	3A9	
CCR6	BV421	11A9	
CD14	BV605	M5E2	
CD20	BV605	2H7	
CD28	PE-Cy7	CD2-28.2	
CD3	PerCP-Cy5.5	SP-34-2	
CD4	FITC	L-200	
CD45	APC-Cy7	D058-1283	
CD8	Alexa700	SK-1	
CD95	PECF594	DX2	
CXCR3	PE	1C6	
Fc Block	N/A	N/A	
Live/Dead Fixable	Yellow	N/A	

#### Peripheral blood mononuclear cell isolation

A number of panels were performed using PB instead of WB samples. The following protocol was used for PB isolation. First, WB samples were centrifuged at 400 × g for 5 minutes, and the plasma was removed. The blood pellet was resuspended in 1 volume of PBS. The manufacturer’s suggested protocol was followed for using the Lymphoprep gradient. The PB were then washed two times in sterile PBS. Depending on the amount of RBC contamination, cells were resuspended in 1–5 ml of ACK lysis buffer, incubated at RT for 5–10 mins, and washed in PBS at 400 × g for 5 min and resuspended in 1 ml PBS. The cells were then enumerated on a Countess II fluorescent cell counter and transferred to appropriate tubes with specific amounts based on the panel performed.

#### Staining procedure for panels C, D, I, M, L (E23), panel AD (E24, E25), panel AA, S (E25)

First, PMBCs were isolated following the protocol described above. The pellet was resuspended in the appropriate amount of antibody master mix as indicated in Table 10, Tables 13, 14, Table 11, and Table 12 for panels C & D, L, M, AA, and S, respectively, and incubated on ice for 45 minutes. 2 ml of sterile 1X PBS was added and washed by centrifuging at 800 × g for 5 min at 4 °C. The cell pellet was resuspended in 1 ml of BD Lyse-Fix PhosFlow and stored at 4 °C overnight or for at least 1 h. Tubes were centrifuged at 800 × g for 5 min at 4 °C to pellet cells followed by washing in 2 ml 1X PBS by centrifugation at 800 × g for 5 min at 4°C. Cells were resuspended in exactly 190 µl of PBS and 10 µl of Life Trucount Beads were added.

**Table 13:** Flow cytometry panel L.

Antibody/probe	Fluorochrome	Clone
CD14	BV605	M5E2
CD183 (CXCR3)	APC	1C6
CD195 (CCR5)	PE	3A9
CD196 (CCR6)	BV421	11A9
CD20	BV605	2H7
CD28	PE-Cy7	CD2-28.2
CD3	PerCP-Cy5.5	SP-34-2
CD4	FITC	L-200
CD45	APC-Cy7	D058-1283
CD8	Alexa700	SK-1
CD95	PECF594	DX2
Fc Block	N/A	N/A
Live/Dead Fixable	Yellow	N/A

**Table 14:** Flow cytometry panel M.

Antibody/probe	Fluorochrome	Clone
BETA7	PE	FIB504
CD14	BV605	M5E2
CD20	BV605	2H7
CD28	PE-Cy7	CD2-28.2
CD3	PerCP-Cy5.5	SP-34-2
CD4	FITC	L-200
CD45	APC-Cy7	D058-1283
CD8	Alexa700	SK-1
CD95	PECF594	DX2
CXCR5	eFluor450	MU5UBEE
Fc Block	N/A	N/A
Live/Dead Fixable	Yellow	N/A

For panel I (E23) and panel AD (E24, E25), the staining procedure was two-step. First, the IgG was prepared in a separate cocktail with FC Block and added for surface IgG staining, incubated on ice for 30 minutes. The cells were then washed with 2 ml of 1X PBS by centrifugation at 800 × g. For the second step, the steps described in the previous paragraph were followed after the cells were resuspended in a cocktail containing the rest of the antibodies as indicated in Tables 15 and 16 for panel I and panel AD, respectively.

**Table 15:** Flow cytometry panel I.

Antibody/probe	Fluorochrome	Clone	Remarks
CD138	PerCP-Cy5.5	M115	
CD14	BV605	M5E2	
CD19	APC-Alexa700	J3-119	
CD20	BV650	2H7	
CD21	PECF594	B-LY4	
CD27	PE-Cy7	M-T271	
CD3	BV605	SP-34-2	
CD45	APC-Cy7	D058-1283	
Fc Block	N/A	N/A	step 1 and step 2
IgD	PE	2030-02	
IgG	Alexa647	4700-31	step 1 only
IgM	Alexa488	2020-30	
Live/Dead Fixable	Yellow	N/A	
PD-1	BV421	EH12.1

**Table 16:** Flow cytometry panel AD.

Antibody/probe	Fluorochrome	Clone	Remarks
CD14	Pacific Blue	M5E2	
CD19	APC-Alexa700	J3-119	
CD20	BV650	2H7	
CD21	PECF594	B-LY4	
CD27	PE-Cy7	M-T271	
CD3	PerCP-Cy5.5	SP-34-2	
CD45	APC-Cy7	D058-1283	
Fc Block	N/A	N/A	Step 1 and Step 2
IgD	PE	2030-02	
IgG	Alexa647	4700-31	Step 1 only
IgM	Alexa488	2020-30	
Live/Dead Fixable	Yellow	N/A	

#### Staining procedure for panels J, N, AE (E23, E24, E25)

First, PMBCs were isolated following the protocol described above. The cells were surface-stained with the antibody cocktail shown in Tables 17–19 for panels J, N, and AE, respectively, followed by incubation overnight at 4 °C in eBioscience FoxP3 fix perm buffer (ThermoFisher) for intracellular markers. After overnight fixing, the cells were washed using standard manufacturer’s procedures and incubated at 4 °C for 45 minutes with antibodies against intracellular markers. Finally, the cells were washed twice in the fix/perm buffer and resuspended in 100–200 μl of PBS.

**Table 17:** Flow cytometry panel J.

Antibody/probe	Fluorochrome	Clone	Remarks
CD138	PerCP-Cy5.5	M115	
CD14	BV605	M5E2	
CD19	APC-Alexa700	J3-119	
CD20	BV650	2H7	
CD21	PECF594	B-LY4	
CD27	PE-Cy7	M-T271	
CD3	BV605	SP-34-2	
CD38	FITC	AT-1	
CD45	APC-Cy7	D058-1283	
Caspase-3	Alexa647	C92-605	Intracellular
Fc Block	N/A	N/A	
IgD	PE	2030-02	
KI-67	BV421	B56	Intracellular
Live/Dead Fixable	Yellow	N/A

**Table 18:** Flow cytometry panel N.

Antibody/probe	Fluorochrome	Clone	Remarks
CD14	BV605	M5E2	
CD20	BV605	2H7	
CD28	PE-Cy7	CD2-28.2	
CD3	PerCP-Cy5.5	SP-34-2	
CD4	FITC	L-200	
CD45	APC-Cy7	D058-1283	
CD8	Alexa700	SK-1	
CD95	PECF594	DX2	
Caspase-3	PE	C92-605	Intracellular
Fc Block	N/A	N/A	
KI-67	Alexa647	B56	Intracellular
Live/Dead Fixable	Yellow	N/A	
PD-1	BV421	EH12.2H7

**Table 19:** Flow cytometry panel AE.

Antibody/probe	Fluorochrome	Clone	Remarks
CD14	BV605	M5E2	
CD19	APC-Alexa700	J3-119	
CD20	BV650	2H7	
CD21	PECF594	B-LY4	
CD27	PE-Cy7	M-T271	
CD3	PerCP-Cy5.5	SP-34-2	
CD38	FITC	AT-1	
CD45	APC-Cy7	D058-1283	
Caspase-3	Alexa647	C92-605	Intracellular
Fc Block	N/A	N/A	
IgD	PE	2030-02	
KI-67	BV421	B56	Intracellular
Live/Dead Fixable	Yellow	N/A	

#### Immunology (cytokine assay: E04, E23, E24, E25)

For the cytokine assay, cryopreserved plasma from lymphoprep (CR) samples for E04 and undiluted cryo plasma samples for E23, E24, and E25 derived from WB collected from the NHPs on major TP days were used. A custom NHP 45-Plex kit from Affymetrix was used following the vendor’s SOP for sample processing and data collection. An LX100/LX200 Instrument was used for data acquisition, and data were analysed in Procarta Plex Analyst software. The following cytokine targets were measured:

NGF-β, CXCL13, Eotaxin, FGF-2, G-CSF, GM-CSF, Granzyme B, IFNα, IFNγ, IL-1β, IL-1RA, IL-10, IL-12p40, IL-12p70, IL-13, IL-15, IL-17A, IL-17F, IL-2, IL-23, IL-4, Il-5, IL-6, IL-7, IL-8, IP-10, ITAC, MCP-1, MCP-3, MIF, MIG, MIP-1α, MIP-1β, PDGF-BB, RANTES, sCD40L, SDF-1α, sICAM-1, TGFα, TGFβ, TNFα, TNFβ, TRAIL, sVCAM-1, VEGF-A.

#### Immunology (ELISA assay: E04)

Erythropoietin (EPO) levels were determined by using a Quantikine IVD ELISA assay for human EPO using the manufacturer’s suggested protocol. All samples were randomized prior to performing the ELISA.

#### Immunology (ELISA assay: E23, E24, E25)

Infected RBC lysates were generated from RBCs collected from two *P. cynomolgi* infected donor monkeys (RQv9 infected with the Ceylon strain and RAg15 infected with the M/B strain). For uninfected (u)RBC assays, separate uRBC lysates were generated from uninfected donor monkeys. The list of 16 assays performed is included below. However, not all samples were used in every assay; see usage notes for more details.

Total IgG, Total IgM, iRBC IgM – M/B strain, uRBC IgM – M/B strain, iRBC IgM - Ceylon strain, uRBC IgM - Ceylon strain, iRBC IgG – M/B strain, uRBC IgG – M/B strain, iRBC IgG - Ceylon strain, uRBC IgG - Ceylon strain, iRBC IgG1 – M/B strain, iRBC IgG2 – M/B strain, iRBC IgG3 – M/B strain, iRBC IgG1 - Ceylon strain, iRBC IgG2 - Ceylon strain, iRBC IgG3 - Ceylon strain.

#### Total IgG and IgM plasma concentration

For measuring Total IgG plasma concentration, Corning high-binding microtiter plates coated with Anti-Monkey IgG + IgA + IgM (Rockland Immunochemicals) diluted in ELISA coating buffer (Abcam) to 0.6 μg/ml were used. The plate was incubated overnight at 4 °C and then washed four times with PBS-T. The plate was blotted dry and blocked using serum-free Sea Block (Abcam) for two hours at RT then washed four times in PBS-T. Plasma samples were diluted to 1:100,000 in 10% Sea Block and were added to each well, followed by an incubation period of 2 h at RT and then washed four times with PBS-T. The plate was blotted dry, and HRP-conjugated anti-IgG (Jackson Immunoresearch) diluted 1:30,000 in 10–33% Sea Block in PBS was added to each well and incubated for 1 h at RT in the dark. The plate was washed four times with PBS-T after incubation, followed by the addition of 100 μl TMB substrate (Abcam), which was allowed to develop for 3–5 minutes. 100 μl Stop solution was added, and the absorbance at 450 nm was measured. Total IgG concentrations were then calculated using Rockland IgG Monkey Calibrator standard based on a 4-PL standard curve.

For Total IgM, the same steps were followed with the following differences. Plates were coated with Anti-Monkey IgM (Life Diagnostics) diluted to 5 μg/ml. Plasma samples were diluted to 1:10,000 instead, and HRP-conjugated anti-IgM (Jackson Immunoresearch) diluted to 1:20,000 was used as the secondary antibody. Finally, a Monkey Total IgM ELISA kit from Abcam was used as the standard.

#### Infected and uninfected RBC specific IgG and IgM concentrations

Uninfected RBC (uRBC) lysates, as well as infected RBC lysates (iRBC) generated from RBCs collected from infections of two donor monkeys (RQv9 for Ceylon strain and RAg15 for M/B strain), were obtained as described in the dataset SOP and Joyner at al.^18^,. iRBC and uRBC specific IgG and IgM concentrations were measured using the same steps as the total IgG and IgM assays described above with the following differences. The plates were coated with iRBC or uRBC lysate diluted in ELISA coating buffer (Abcam) to 5 μg/ml. Plasma samples were diluted to 1:100 instead.

Finally, parasite specific IgG subclass concentrations were measured by the following additional steps to the protocol described above. After the plate was coated with iRBC lysate, duplicate wells were coated with recombinant expressed rhesus IgG1, IgG2, or IgG3 from NHP Reagent Resource diluted to 1 μg/ml in ELISA coating buffer before incubation. Additionally, mouse anti-rhesus IgG1, IgG2, or IgG3 from NHP Reagent Resource diluted 1:10,000, 1:1,000, and 1:10,000 in 10% Sea Block in PBS, respectively, was added to each well and incubated at RT for 1 h before the HRP-conjugated anti-mouse IgG from Jackson Immunoresearch diluted 1:10,000 in 10% Sea Block in PBS was added.

#### Transcriptomics (RNA-Seq: E04)

WB (3 ml) was collected from each animal at TPs in Tempus tubes (Applied Biosystems), which preserve the integrity of mRNA. Samples included erythrocytes, platelets, and granulocytes in addition to mononuclear lymphocytes. WB RNA was extracted using Tempus-Spin RNA isolation kits.

BM (1 ml) was collected from each animal at TPs into 1.5 ml tubes with EDTA, and the mononuclear cells were purified by density gradient centrifugation on Lymphoprep (Stem Cell Technologies) solution and preserved in RLT buffer (Qiagen) to stabilize the mRNA. RNA was extracted from the BM using Qiagen RNeasy Mini-Plus kits following the manufacturer-recommended procedures, and from PB samples using Tempus-Spin RNA isolation kits.

Approximately 1 μg of total RNA per sample was converted to double-stranded cDNA using poly-A beads to enrich for mRNA, and Illumina TruSeq Stranded mRNA Sample Prep kits to generate strand-specific libraries. Adapters were ligated to facilitate 3-plex sequencing on an Illumina HiSeq. 1000 at the Yerkes Genomics Core, aiming for 80 million paired-end 100 base pair (bp) reads per library.

RNA was sequenced by the Yerkes Genomics Core. The sequence data (fastq files) were mapped by the MaHPIC’s Transcriptomics/Functional Genomics core to the *M. mulatta* and *P. cynomolgi* reference genomes^22,23^. The mapping results were used to generate expression profiles for genes across all TPs, NHPs, and specimen types. The dataset consists of sequence data, alignment files, raw count and normalized expression tables, related metadata, data production and analysis SOPs, and a detailed README.

Bases were called with Illumina RTA (Real-Time Analysis, v1.13.48) with default parameters. FASTQC (v0.10.1) was used to assess data quality, but the data were not filtered at this stage. To quantify gene expression, RNA-Seq reads were mapped to the listed assembly and annotation using Tophat2^24^. Default options were used with the exception that the command-library-type fr-secondstrand was used since reads were generated using a stranded library preparation method from Illumina. This allowed differentiation between sense and antisense transcripts. Only reads that map to a single location in the genome were included, to ensure high-confidence mapping. Transcript abundance levels were inferred using HTSeq v0.5.4 (http://www-huber.embl.de/users/anders/HTSeq/doc/). HTSeq takes the short-read mapping file (bam) from Tophat2 and the gene annotation file which contains the locations of all annotated genes. Since some libraries were sequenced more deeply than others, the libraries were normalized before determining differential gene expression using the gene level expression files with the default parameters of DESeq v1.10.1 (http://www.bioconductor.org/packages/release/bioc/html/DESeq.html).

RNA-Seq reads were mapped to both the host and parasite genome. For host, an early version of a new assembly (as of 5/2014) was used, of the rhesus macaque/*M. mulatta* MacaM assembly, v4.0, created by Aleksey Zimin at the University of Maryland, Rob Norgren at the University of Nebraska Medical Center and their colleagues. The MacaM assembly has been deposited in GenBank under accession PRJNA214746 ID: 214746. For the parasite, PlasmoDB version 9.3 of the *P. cynomolgi* M/B strain genome assembly was used. The assembly was deposited in GenBank under the accession PRJDA49901 ID: 49901.

E04 WB and BM samples were submitted separately to NCBI GEO and SRA). The Results excel files contain normalized transcript abundances at the gene level, for each sample TP, from each individual. Abundances are further classified by experimental TP and specimen type. Note that overlapping genes (sharing exons in the annotation) were collapsed into a single gene for the purposes of RNA-Seq read assignments. So, the read count at such a locus is representative of the cumulative expression of all the genes at that locus, but the entire read count is assigned to only one of the genes and the others in the locus are assigned a ‘0’.

Gene data column headers are defined as follows:

- ‘gene_name’: Identifiers of all genes in the reference annotation.
- ‘gene_symbol’: Symbols of all genes in the reference annotation.
- ‘Sample ID/Raw File 1/Raw File 2 /Normalized Read Counts’: Sample ID, Raw sequence file names for the sample, and Library size normalized and Log2 transformed read counts of all genes, from the Specimen Type, Individual ID, and TP listed directly below.

#### Transcriptomics (RNA-Seq: E04R)

Most E04 sample collection and analysis methods also apply with E04R. Distinct methods are as follows. Sequencing was done on an Illumina HiSeq. 3000. Bases were called with Illumina RTA (Real-Time Analysis, v2.7.7) with default parameters. Illumina bcl2fastq v2.17.1.14 was used for demultiplexing. FASTQC (v0.10.1) was used to assess data quality, but the data were not filtered at this stage. Reads were mapped to a composite reference assembly consisting of host, parasite, and External RNA Controls Consortium (ERCC) control references with STAR (v2.5.2b) with default alignment parameters (to the same version of the host and parasite genome assembly and annotation in E04). Abundance estimation of raw read counts per transcript was done internally with STAR using the algorithm of HTSeq-count. Normalized expression (normalized read counts) was performed with DESeq. 2 (v1.10.1). The Results excel files contain either normalized transcript abundances or raw counts at the gene level, for each sample TP, from each individual. Note that there is a read count entry per sample for every gene that appears in the annotation. For genes where there was no detection of expression by reads that mapped to their loci, the raw count is ‘0’.

Gene data column headers are defined as follows:

- ‘Gene ID’: Identifiers of all genes in the annotation.
- ‘Gene Symbol’: Symbols of all genes in the annotation.
- ‘Raw File List/Sample Identifier/Abundances’: Samples were sequenced across multiple lanes, some samples were sequenced at extra depth, all fastq files are listed for each sample. DESeq. 2 normalized read counts of all genes, from the raw files listed in the column header. The raw file names and sample identifier both contain information regarding the specimen type, each individual NHP ID code, and TP.

#### Transcriptomics (RNA-Seq: E23R, E24, E25)

WB and BM sample collection, RNA extraction, double-stranded cDNA conversion, library preparation, and sequencing were performed as for E04R with the exception that adapters were ligated to facilitate multiplexed sequencing on an Illumina HiSeq. 3000 at the Yerkes Genomics Core, aiming for 50 million paired-end 100 base pair (bp) reads per library.

Base calling, quality assessment, quantification, mapping (to the same version of the host and parasite genome assembly and annotation in E04), and the calculation of abundance levels were performed as for E04R with the following exceptions.

- E23R, E24, and E25 datasets do not contain normalized results.
- No spike-ins were used for E24 or E25 samples.

The dataset consists of sequence data, alignment files, raw count expression tables, related metadata, data production and analysis SOPs, and a detailed README. Results file formats are the same as for E04R.

#### Transcriptomics (RNA-Seq: E13)

WB and BM collection, RNA extraction, double-stranded cDNA conversion, library preparation, sequencing, base calling, quality assessment, quantification, mapping (to the same version of the host genome assembly and annotation), and the calculation of abundance levels were performed as for E04. Distinct methods are as follows.

The dataset consists of sequence data, normalized abundance counts, related metadata, and a detailed README. Additional details are provided at NCBI (BioProject, SRA, and GEO sites, see the Data Records section). The Results excel files contain normalized transcript abundances, separately at the gene and exon levels, for each individual. Abundances are further classified by experimental TP and specimen type. Note that overlapping genes (sharing exons in the annotation) were collapsed into a single gene for the purposes of RNA-Seq read assignments. So, the read count at such a locus is representative of the cumulative expression of all the genes at that locus, but the entire read count is assigned to only one of the genes, and the others in the locus are assigned a ‘0’.

Gene Data Column headers are defined as follows:

- ‘gene_name’: Identifiers of all Genes in the reference annotation.
- ‘gene_symbol’: Symbols of all Genes in the reference annotation.
- ‘Sample ID/Raw File 1/Raw File 2 /Normalized Read Counts’: Sample ID, Raw sequence file names for the sample, and Library size normalized and Log2 transformed read counts of all genes, from the Specimen Type, Individual ID, and TP listed directly below.

Exon Data Column headers are defined as follows:

- ‘Exon ID’: IDs were generated during analysis. Each row contains the Exon Identifier of one exon, for which expression data were recorded. IDs are composed of two parts separated by a colon. The first part is the symbol of the gene to which this exon belongs. The second part is a numerical identifier for the order of this exon in the listed gene.
- ‘Start Location of Exon’: Each row contains start location of one exon. Note that this location may not match the start location in the original annotation. See Usage Notes.
- ‘End Location of Exon’: Each row contains end location of one exon. Note that this location may not match the end location in the original annotation. See Usage Notes.
- ‘Strand of Exon’: Each row contains strand information of one exon.
- ‘Gene ID’: Each row contains the Gene Identifier of the gene for that exon. Note that only one gene ID is listed even when the exon is shared among multiple genes. See Notes section.
- ‘Gene Symbol’: Each row contains the Gene Symbol of the gene that the exon belongs to. Note that only one Gene Symbol is listed even when the exon is shared among multiple genes.
- ‘Gene and Transcript Membership of Exon’: Each row contains all the genes and transcripts that one exon belongs to (has membership in). This column provides the useful information to identify the exons, as they are listed in the reference annotation.
- ‘Sample ID/Raw File 1/Raw File 2/Normalized Read Counts’: Sample ID, Raw sequence file names for the sample, and library size normalized and Log2 transformed read counts of all exons. The column header also contains information regarding Specimen Type, Animal ID, and TP. Each row contains the library size normalized and Log2 transformed read count observed for one exon, in the condition/sample.

#### Metabolomics (LC-MS/MS: E13, E04, E23)

Dataset results include *m/z* (mass-to-charge), retention times,, and ion intensities for every sample produced by the MaHPIC Metabolomics core at Emory University using established methods as described in the dataset SOPs and in Uppal, *et al*.^25^. The dataset contains extensive documentation of SOPs and workflows, including software parameters, sample and QC sample preparation, LC-MS conditions, *in silico* analyses, and results generation. All files are described in the dataset README.

Samples were also collected during the experimental daily follow up and assigned TPs. After capillary or venous EDTA samples had been processed for clinical assays, any remaining volume was transferred to a microcontainer tube and centrifuged at 2000 rpm for 10 minutes at room temperature. Plasma was aliquoted into Eppendorf tubes and stored at −80 °C.

All samples were randomized to prevent batch degradation in any one set of samples. Individual subjects were grouped separately and then randomized within the subject group. Example of a batch: Technical triplicates = 20 samples + 2 qstd = 25 hours (6 injections total).

Samples were analysed in an untargeted high-resolution metabolomics LC-MS assay using a Dionex Ultimate 3000 LC coupled with Thermo High Field QExactive for E13 and E23 and QExactive for E04. Each sample was run in triplicate with 5–10 µl injection volume on each column with the following optimized settings:

- Positive mode (AE for E13, C18 for E04 & E13, HILIC for E23): HESI probe with S-lens combination for ESI; MS1 mode scanning *m/z* range of 85–1275; Resolution – 70,000 (E04), 120,000 (E13, E23); maximum number of ions collected – 1 × 106; The maximum injection time – 200; capillary temperature - 300 °C; Aux gas heater temp - 200 °C; spray voltage – 3.5 kV; sheath gas – 45; Auxiliary gas flow – 25; sweep gas flow – 1; S-lens RF level – 69
- Negative mode (C18): HESI probe with S-lens combination for ESI; MS1 mode scanning *m/z* range of 85–1275; Resolution – 70,000 (E04), 120,000 (E13, E23); maximum number of ions collected – 1 × 106; The maximum injection time - 200; capillary temperature - 300 °C; Aux gas heater temp - 200 °C; spray voltage – 3.0 kV; sheath gas – 45; Auxiliary gas flow – 25; sweep gas flow – 1; S-lens RF level – 69

“.raw” files were first converted to “.cdf” files using Thermo file converter which were subsequently processed using apLCMS (v5.9.4 for E13, v6.0.1 for E04, v6.3.3 for E23)^26^. apLCMS is a set of algorithms written to work with the statistical R program. In this set of algorithms, large capacities of LC-MS data can be analysed using the cdf.to.ftr() wrapper function which performs noise removal, peak detection, *m/z* and retention time alignment, and quantification.. The output of apLCMS includes a feature table where the rows are features (defined as the unique combination of *m/z* and retention time) that appeared in the LC-MS run, and the columns include the ion intensities of each of these features for every sample.

All batches were then run together as a whole with xMSanalyzer (v1.3.3 for E13, v2.0.2 for E04, v2.0.7 for E23)^25^. The R script ran through multiple parameter settings. xMSanalyzer merged different parameter settings and discarded anything with a CV of higher than 50% and generated sample and quality reports. This information was included in the feature table worksheet. Batch-effect correction is performed using ComBat^27^ implemented in the “sva” package in R Bioconductor. PCA plots are generated to verify. Finally, annotation was performed using xMSannotator (v1.3.2 for E13, v1.2.1 for E04, v1.3.2 for E23)^28^ against HMDB^29^ (v3.6 for E13 & E23, v3.5 for E04). Alternative annotations against KEGG^30^ release v71.1 are also available for E13 and E04 datasets.

#### Lipidomics (LC-MS/MS: E04, E23, E25)

E25 lipidomics used only WB samples, otherwise this section applies to all three experiments. Dataset results consist of relative quantification results of targeted lipid species, raw and intermediate data, related metadata, and supporting documents from the MaHPIC Lipidomics core at Washington University in St. Louis, MO. The dataset contains extensive documentation of SOPs and workflows, including software parameters, sample and QC sample preparation, LC-MS/MS conditions, *in silico* analyses, calculation of peak areas, and results generation.

Multiple targeted lipid classes were quantified for E04, E23, and E25:

- Glycerophospholipids - phosphatidic acid, phosphatidylcholine, phosphatidylserine, phosphatidylethanolamine, phosphatidylinositol
- Sphingolipids - ceramide, sphingomyelin, glucosylceramide, lactosylceramide (WB only), sulfatide, ganglioside GM3
  Additional targeted lipid classes were quantified for E25:
- Glycerolipids – diacylglycerol (BM only)

WB and BM samples were prepared by combining 20 µL of a sample with 40 µL of water in a 2 mL VWR tube. The samples were vortexed for one minute. 50 µL of internal standard and 0.3 mL of methanol were added, and the sample was vortexed for three minutes. The samples were centrifuged at 10,000 rpm, and the supernatant transferred to a 1.2 mL glass insert, dried with nitrogen at 50 °C, reconstituted with 200 µL of methanol-chloroform (9:1), centrifuged 3750 rpm for five minutes, and the supernatant transferred to a clean 1.2 mL glass insert.

Lipids were detected in selected reaction monitoring (SRM) or multiple reaction monitoring (MRM) modes. The dataset SOP describes the preparation of all internal standards. A quality control (QC) sample was prepared by pooling the extracts from the same sample as the study samples, and aliquoted and stored at −80 °C (additional QC sample preparation details are present in the dataset SOPs). The QC sample was injected 6 times at the beginning of each LC-MS/MS run to stabilize the instrument, and then every 5 study samples to monitor the LC-MS/MS performance in each batch. Prior to sample extraction, the samples were randomized via blind drawing.

Single injections were made for all experimental samples. Four LC-MS/MS runs were used to analyse all lipids. Specific lipids for each run, HPLC flow rate tables, SRM tables, and MRM tables are provided in the dataset SOPs. Two instruments were used for mass spectrometry, AB Sciex API4000 and Thermo Scientific TSQ Quantum Ultra (TSQ). Each was used to detect specific lipid classes in specific sample types. Note that BM results do not include Lactosylceramide and WB results do not include Diacylglycerol.

- BM with API4000: ganglioside GM3 and sulfatide
- BM with TSQ: ceramide, diacylglycerol, glucosylceramide, phosphatidic acid, phosphatidylcholine, phosphatidylethanolamine, phosphatidylinositol, phosphatidylserine, sphingomyelin
- WB with API4000: ganglioside GM3 and sulfatide
- WB with: ceramide, glucosylceramide, lactosylceramide, phosphatidic acid, phosphatidylcholine, phosphatidylethanolamine, phosphatidylinositol, phosphatidylserine, sphingomyelin

HPLC conditions for phosphatidylcholine, sphingomyelin, ceramide, phosphatidylserine, phosphatidylinositol, phosphatidic acid, phosphatidylethanolamine, glucosylceramide, diacylglycerol and lactosylceramide were as follows:

- Equipment - Shimadzu 10ADHPLC system with a CBM-20A system controller, 2 LC-10AD pumps, a SIL-20AC autosampler
- Mobile phase A: 10 mM ammonium acetate in acetonitrile-water (3:7)
- Mobile phase B: 10 mM ammonium acetate methanol-isopropanol (1:1)
- Guard column: SecurityGuard C18 (4 × 3 mm)
- Analytical column: XBridge C18 (3 × 100 mm, 3.5 µm)
- Column temperature: 50 °C
- Injection volume: 8 µL (phosphatidylcholine and sphingomyelin) 10 µL (ceramide, diacylglycerol, phosphatidylserine, phosphatidylinositol, phosphatidic acid, phosphatidylethanolamine, glucosylceramide and lactosylceramide)

HPLC conditions for sulfatide and ganglioside GM3 were as follows:

- Equipment - Shimadzu 20 ADHPLC system with a CBM-20A system controller, 4 LC-20AD pumps, a CTC PAL autosampler, and a Valco 6-port switching valve
- Mobile phase A: 10 mM ammonium fluoride in acetonitrile-water (3:7)
- Mobile phase B:10 mM ammonium fluoride in methanol-isopropanol (1:1)
- Mobile phase C: 1.5% formic acid in water
- Mobile phase D: acetonitrile
- Trapping column: Thermo-Keystone C-18 (4 × 10 mm, 5 µm)
- Analytical column: Xselect HSS C18 (4.6 × 100 mm, 3.5 µm)
- Column temperature: room temperature
- Injection volume: 30 µL

TSQ mass spectrometric conditions were as follows:

- Polarity: negative (phosphatidylcholine and sphingomyelin), positive (ceramide, diacylglycerol, phosphatidylserine, phosphatidylinositol, phosphatidic acid, phosphatidylethanolamine, glucosylceramide and lactosylceramide)
- Ion source: electrospray
- Capillary temperature: 250.0
- Vaporizer temperature: 250.0
- Sheath gas pressure: 60.0
- Ion sweep gas pressure: 0.0
- Aux valve flow: 20.0
- Spray voltage: −4000 (phosphatidylcholine and sphingomyelin), 4000 (ceramide, diacylglycerol, phosphatidylserine, phosphatidylinositol, phosphatidic acid, phosphatidylethanolamine, glucosylceramide and lactosylceramide)
- Method Type: EZ Method
- MS Run Time (min): 24.00
- Experiment Type: SRM
- Chromfilterpeak width (s): not used
- Collision gas pressure (mTorr): 1.5
- Use tuned tube lens value: Yes
- Q1 peak width (FWHM): 0.70
- Display time range for SRM table: No
- Skimmer offset (V): 10
- Scan time (second): 0.02 per transition

API4000 mass spectrometric conditions were as follows:

- Scan Type: MRM
- Scheduled MRM: No
- Polarity: Negative
- Scan Mode: N/A
- Ion Source: Turbo spray
- Resolution Q1: Low
- Resolution Q3: Low
- Intensity Thres.: 0.00 cps
- Settling Time: 0.0000 msec
- MR Pause: 5.0070 msec
- MCA: No
- Step Size: 0.00 Da
- CUR: 20.00
- GS1: 40.00
- GS2: 58.00
- TEM: 450.00
- ihe: ON
- CAD: 7.00
- IS: −4200.00
- EP: −10.00
- Dwell time (ms): 20 per transition

#### Lipidomics (LC-MS/MS: E13)

Dataset results contain quantitative analysis of 5 classes of long chain bases (sphingolipids: sphingosine, sphingosine-B, sphinganine, sphingosine phosphate, sphinganine phosphate)^31,32^, raw data (both instrument and analysed results files), related metadata, and supporting documents from the MaHPIC Lipidomics core at the YNPRC of Emory University. The dataset contains extensive documentation of SOPs and workflows, including software parameters, sample and QC sample preparation, LC-MS/MS conditions, *in silico* analyses, calculation of peak areas, and results generation.

Sphingolipids were extracted from WB samples and prepared for LC-MS/MS analysis of sphingoid and sphingolipid fractions as follows: An internal standard mix (see dataset SOPs), a complex sphingolipid internal standard mixture, and a calibrator solution were prepared (see dataset SOPs). Samples or calibrator mixture was added to designated tubes (hereafter referred to as samples). 0.5 mL methanol, and 0.25 mL chloroform were added to all samples. Tubes were sonicated for 1 minute. All samples were incubated at 48 °C in a heating block overnight and cooled to room temperature. 75 µL of 1 M potassium hydroxide in methanol were added to all samples which were then vortexed briefly and sonicated for 1 minute. Samples were incubated for 2 hours at 37 °C in a shaking water bath and then centrifuged for 30 minutes at 3000 rpm. 400 µL of supernatant was transferred to new labeled tubes and dried under nitrogen at 37 °C to prepare samples for LC-MS/MS sphingoid base analysis.

The remaining liquid in each original sample tube was further processed by adding 3 µL of glacial acetic acid and then vortexing for 1 minute. 1 ml chloroform and 2 mL water were added to all samples, and samples were vortexed for 1 minute before centrifuging at 4000 g for 10 minutes. The upper aqueous layer was removed, and the samples were dried under nitrogen at 37 °C to prepare samples for LC-MS/MS analysis of the complex sphingolipid fraction.

Lipid extracts were analysed by LC-MS/MS with an AB Sciex 6500 triple quadrupole mass spectrometer using MRM (multiple reaction monitoring) in positive ionization mode coupled to a Shimadzu HPLC. Internal standards were injected 14 times at the beginning of each LC-MS/MS run. One or more blanks were injected between all standards and samples. Each sample was injected in two replicates. At the start of the run, all solvent lines were purged and a Phenomenex Synergi Fusion-RP column was attached. All experimental and calibrator samples were resolubilized in 300 µL of 1:1 methanol/water, vortexed and sonicated for 1 minute before being centrifuged for 5 minutes at 12300 rpm. The supernatant from each sample was transferred to autosampler vials and placed in the autosampler tray. The Analyst software (Sciex, version 1.6) was used for to submit the batch for analysis, and the instrument was equilibrated instrument for 10 minutes. Raw instrument files were stored in.wiff format.

Data were interpreted using the Analyst software Quantitation Wizard. Output files were stored in.rdb format. Results were transferred to excel with one worksheet for each class of lipid analysed. Excel results contain peak names, peak areas, peak heights, concentration of standards, and a measure of accuracy based on the known concentrations of standards.

#### Proteomics (E04, E23)

The Complex Carbohydrate Research Center (CCRC) Proteomics core at the University of Georgia received prepared samples of isolated RBC membranes for LC-MS/MS analysis. Spectral count normalization was recorded in output files. Raw data is provided for users who wish to implement alternative normalization methods post-processing. Intermediate results are provided for users who wish to perform analyses with different tools and/or parameters. Data was normalized via ProteoIQ (PREMIER Biosoft) using ‘separate sampling + NSAF'^33^. All runs were combined into a single result. The CCRC proteomics core further ‘certified’ those proteins identified in at least 50% of NHPs for any single TP or in 50% of TPs. The resulting sequence data were mapped to validated *M. mulatta* and *P. cynomolgi* reference genome sequences (Uniprot, NuSep Inc.). The dataset contains extensive documentation of SOPs and workflows, including software parameters and screenshots of software configuration, for sample processing, *in silico* analyses, and results generation. Samples were thawed on ice, and 400 µl were added to prepared collection tubes using C18 cartridges (Nest Group, MacroSpin Column – SMM SS18V, one cartridge per sample, cartridges were prepared by washing three times each with 450 µl of 100% methanol, 100% Acetonitrile (ACN), 80% ACN in 0.1% Formic acid (FA), 0.1% FA spun at 500 g for 3 minutes, flowthrough discarded). Samples were spun at 500 g for 3 minutes and reapplied to the cartridge once before discarding flowthrough. Each cartridge was washed with 450 µl of 0.1% FA, spun at 500 g for 3 minutes, and the flowthrough discarded (repeated for 4 times total). Each cartridge was moved to a fresh collection tube, and 300 µl of 80% ACN in 0.1% FA was added to elute peptides. Tubes were spun at 500 g for 3 minutes (repeated for 2 times total), and peptides were transferred to a final fresh tube. The samples were concentrated in a speed vacuum.

Reverse phase nanoLC-MS/MS (RPnanoLCMSMS) was performed on an Orbitrap Fusion™ Tribrid™ Mass Spectrometer (Thermo Fisher Scientific). Dried pellets were resuspended in 0.5 µl of 0.1% FA/80% ACN and 19.5 µl of – 0.1% FA. Peptides were passed through a 0.22 um spin filter (Pall ODM02C35) and transferred into autosampler vials. Samples were injected with an Ultimate 3000 (Thermo Fisher Scientific) autosampler, and gradient separation was performed (see dataset SOP).

The mass spectrometer was operated with standard instrument software (Tune 1.1). The spray voltage was 2.2 kV, and the temperature of the ion transfer tube was 280 °C. Full MS scans were collected from *m/z* 300–2000 at a resolution of 120,000 (FWHM at *m/z* 200), with a maximum ion injection time of 200 ms, and an automatic gain control (AGC) setting of 3E5 ions. MS/MS scans were collected in the ion trap via collision-induced dissociation (CID) using 38% normalized collisional energy, with a maximum injection time of 100 ms for 1 microscan and an AGC setting of 2E4. A dynamic exclusion window was applied to prevent the same *m/z* value from being selected for 30 sec after its acquisition. Data acquisition was conducted using Xcalibur® (ver. 3.0.49, Thermo Fisher Scientific) in the fashion of one Orbitrap full MS followed by top-speed mode (3-sec cycle) data-dependent ion trap CID MS/MS. Each run generated a single Thermo RAW file (.raw)

Sequence databases were created with reference genome sequences and annotations (see E04 Transcriptomics) and known contaminants using NuSep FASTA Manager 1.2.1 and manual formatting of sequence headers in output files. Sequence databases were added to ProValT (v. PROVALT_3.1.05_08-31-16 or greater) (PREMIER Biosoft) and configured. The ProValt functional annotation option was used to add sequence descriptions. The final output sequence database was a concatenation of host, parasite, and contamination sequences. Analysis workflows were created and configured with Proteome Discoverer v2.1 (Thermo Fisher Scientific) and Byonic v2.9 (Protein Metrics Inc.). Proteins were identified using Proteome Discoverer Daemon v2.1 (Thermo Fisher Scientific) and PMI-Batcher (Byonic 2.9, Protein Metrics Inc.). Proteomes were validated using ProValt. Reports of final results were generated with outputs from Proteome Discoverer search results, Byonic search results, ProValt Peptide output files, and raw mass spec files. ProteoWizard was used to create mzXML files.

The Results files include column header definitions for intermediate and final results, including:

- ProteoIQ peptides and proteins
- SC + NSAF Norm results – The entire proteome for that experiment combining all individual results (which were validated at 1% protein FDR). The normalized spectral columns (N-SC) were calculated using 1) Total spectral count for each sample and 2) Normalized spectral abundance factors (NSAF).
- SC + NSAF Norm Top Proteins results – The same data as presented in SC + NSAF Norm results except that only the “Top” proteins are shown.
- SC + NSAF + App Norm results – The entire proteome for that experiment combining all individual results (which were validated at 1% protein FDR). The normalized spectral results were calculated using 1) Total spectral count for each sample, 2) Normalized spectral abundance factors (NSAF), and 3) Apportion shared peptides option
- SC + NSAF + App Norm Top Proteins results – The same data as presented in SC + NSAF + App Norm results except that only the “Top” proteins are shown.

## Data Records

### Data access and reuse

All publicly available files, data, metadata, and results can be accessed (directly or as links to archival repositories NCBI SRA^34^, NCBI BioProject (https://www.ncbi.nlm.nih.gov/bioproject/), GEO^35^, PRIDE^36^, MaSSIVE (https://massive.ucsd.edu/ProteoSAFe/static/massive.jsp), MetaboLights^37^, Metabolomics Workbench (https://www.metabolomicsworkbench.org), and CyVerse Data Commons^38^) from the PlasmoDB^39^ resource, https://plasmodb.org/plasmo/app/static-content/PlasmoDB/mahpic.html. All readers, and especially those interested in reusing MaHPIC data, should consider the clinical datasets as the foundation of each experiment. The clinical dataset information applies to and informs on all other datasets for an experiment. Each public dataset contains a detailed data production and analysis SOPs, metadata for samples and instruments and software, raw instrument data (when applicable), processed results, an explanatory file-naming SOP, and a detailed README that contains information critical to effective data reuse, and any caveats or notes that are considered important for data reuse. Data, metadata, and results were collected in templates designed by the MaHPIC, and for each dataset, specific relevant fields are populated in the templates. The templates include definitions of the fields used, to facilitate comprehension. Files are named in a consistent and structured manner, which provides essential information such as the experiment number, host and parasite species ‘at a glance’. The file-naming SOP is included with each dataset. Controlled versions of reference genome sequences for the *M. mulatta* host and *P. cynomolgi* parasites being studied in E04, E23, E24, and E25 were obtained and validated by the MaHPIC Informatics core. These were made available for project analyses from a central data repository, and versions were controlled within and across experiments to support comparable analyses^9^. To facilitate human and machine readability of file names, and to streamline communication within files a 2-letter naming convention was used for sample types.

### Public data depositions

Data were deposited in public repositories (NCBI SRA^34^, NCBI BioProject, GEO^35^, PRIDE^36^, MaSSIVE, MetaboLights^37^, Metabolomics Workbench and CyVerse Data Commons^38^) or PlasmoDB^39^ (for data types that do not have an archive) with enough information to facilitate findability, individual reuse of data sets, and an understanding of the project breadth. Quality controlled, internally validated datasets (see Technical Validation) were submitted to appropriate public repositories based on their requirements. At the time data were deposited, the metadata requirements for the MaHPIC usually exceeded those of repositories. Custom SOPs were created for each data type to ensure that the entirety of each dataset (SOPs, metadata, comprehensive README files, etc.) were made available in addition to minimum repository requirements. In some cases, the required files not recognized by repositories had to be uploaded as supplementary or accessory files. A dedicated landing page was created to provide a comprehensive overview that shows the relationship of all experiments and datasets (MaHPIC PlasmoDB Landing Page - http://plasmodb.org/plasmo/mahpic.jsp). A summary of samples per NHP for each experiment is available in Supplementary Tables 1–5.

### Clinical, haematological, parasitological data (PlasmoDB)

At the time data were deposited there was no archival repository for MaHPIC’s clinical datasets. Datasets were made available via PlasmoDB^39^. A custom submission template was designed to describe the project, the specific experiment, and all files in each dataset. For each experiment, files were organized into separate folders for results, metadata, and supplementary information. Files organized by experiment can be downloaded at the PlasmoDB landing page and at the links below. To provide Digital Object Identifiers (DOIs) for each dataset, files were also made available at the CyVerse Data Commons^38^ (see citation for each dataset). Datasets contain a detailed README, a single consolidated results file with all measurements for each NHP, SOPs that describe data generation and analysis and metadata collection, and several supplementary files that describe the experiment and provide veterinary and handling details for each NHP (including a record of daily activities for each subject). The contents of all files are described in the README.

- E13 http://plasmodb.org/common/downloads/MaHPIC/Experiment_13/^40^
- E04 http://plasmodb.org/common/downloads/MaHPIC/Experiment_04/^41^
- E23 http://plasmodb.org/common/downloads/MaHPIC/Experiment_23/^42^
- E24 http://plasmodb.org/common/downloads/MaHPIC/Experiment_24/^43^
- E25 http://plasmodb.org/common/downloads/MaHPIC/Experiment_24/^44^

### Immunology (ImmPort)

Within the MaHPIC each type of immunology analysis was part of a separate dataset with its own README that described the files, samples, subjects, etc. This generated separate datasets for innate and adaptive flow cytometry, ELISA, and cytokine assays (see the Detailed Experimental Methods and Data Analyses section). As part of the deposition to ImmPort^45^, MaHPIC datasets were combined into two studies, SDY1015 and SDY1409 (see links below). Within ImmPort, datasets are organized according to their standards into results, protocols, and additional study files and are searchable according to ImmPort’s metadata requirements. All the files from each MaHPIC dataset are included at ImmPort, including the detailed READMEs with file descriptions, to facilitate reassembly of the individual datasets if desired.

- E04 Flow Cytometry - SDY1015^46^

- E04 Cytokine Assay - SDY1015^46^
- E23 Flow Cytometry - SDY1409^47^
- E23 ELISA - SDY1409^47^
- E23 Cytokine Assay - SDY1409^47^
- E24 Flow Cytometry - SDY1409^47^
- E24 ELISA - SDY1409^47^
- E24 Cytokine Assay - SDY1409^47^
- E25 Flow Cytometry - SDY1409^47^
- E25 ELISA - SDY1409^47^
- E25 Cytokine Assay - SDY1409^47^

### Transcriptomics (NCBI BioProject, GEO, SRA)

All transcriptomics results are part of the same NCBI Umbrella BioProject (PRJNA368917^48^) and GEO^35^ Super Series (GSE94274^48^). Both are aggregates of individual BioProjects and GEO series. Each MaHPIC dataset has its own unique accessions ‘under’ these (see links below). Within the MaHPIC, individual datasets were separated based on experiment and sample type and each of these was submitted separately to GEO and the SRA^34^. All files from each MaHPIC dataset are available for download on the GEO series pages for each dataset, including detailed READMEs.

- E13 WB and BM - BioProject PRJNA385821^49^ - GEO Series (70 samples) GSE58340^50^ - SRA Study (70 runs) SRP106798^51^
- E04 BM - BioProject PRJNA385819^52^ - GEO Series (31 samples) GSE94273^53^ - SRA Study (31 runs) SRP106638^54^
- E04 WB - BioProject PRJNA388645^55^ - GEO Series (30 samples) GSE99486^56^ - SRA Study (30 runs) SRP108356^57^
- E04R WB - BioProject PRJNA401436^58^ - GEO Series (33 samples) GSE103507^59^ - SRA Study (33 runs) SRP116793^60^
- E23R WB - BioProject PRJNA412080^61^ - GEO Series (48 samples) GSE104223^62^ - SRA Study (48 runs) SRP118827^63^
- E24 WB - BioProject PRJNA408250^64^ - GEO Series (15 samples) GSE104101^65^ - SRA Study (15 runs) SRP118503^66^
- E25 WB - BioProject PRJNA412382^67^ - GEO Series (25 samples) GSE104330^68^ - SRA Study (25 runs) SRP118996^69^

### Metabolomics (MetaboLights, Metabolomics Workbench)

E13 results were deposited in Metabolomics Workbench. Within Metabolomics Workbench the README and other MaHPIC dataset files are only available for download along with the entire zipped file package. Note that E13 was the first chronological MaHPIC experiment. Subsequent metabolomics results were deposited to MetaboLights^37^, including E04 and E23, according to ISA-TAB requirements that describe the protocols, samples, assays, and metabolites. Both ISA-TAB and the original files from each MaHPIC dataset are available for download. Importantly the MaHPIC README files are included among the raw data files.

- E13 Metabolomics – ST000592^70^
- E04 Metabolomics - MTBLS517^71^
- E23 Metabolomics - MTBLS542^72^

### Lipidomics (MassIVE)

For deposition to MassIVE the MaHPIC files for each dataset were grouped into two folders, ‘raw’ which contains all raw results files, and ‘other’ which contains all other files including the dataset README. Files for each dataset can be downloaded from the ‘Browse Dataset Files’ tab on each record page.

- E13 - MSV000080636^73^
- E04 - MSV000080610^74^
- E23 - MSV000080637^75^
- E25 - MSV000081138^76^

### Proteomics (PRIDE)

Proteomics datasets were deposited at PRIDE^36^ and include raw, intermediate, and final results. Note that the results of E18 (an uninfected control experiment not included in this paper) were included along with other results and files for E04 since they were analysed along with E04. In addition to the main README for each dataset there is a separate README file for intermediate results that details the SOPs used at each stage of analysis (see methods)

- E04 - PXD007774^77^
- E23 - PXD007775^78^

## Technical Validation

### General quality control

The MaHPIC strived to achieve a project mindset and culture of quality control, results validation, development and adherence to SOPs for all stages of data production and analysis, and repeated training on project best practices^9^. Because of the complex, transdisciplinary, and geographically distributed nature of the MaHPIC, a dedicated team was tasked with providing bioinformatics, information technology, and *in silico* quality control for the entire project. This team was known as the Informatics Core, or InfoCore. The InfoCore worked with all institutional staff at all sites and all MaHPIC members to design and implement a barcoded sample tracking system that ensured the correct association of physical samples with downstream aliquots and files, metadata standards (based on existing and emerging standards for each data type), secure data transfer protocols that met both NIH/NIAID contract security requirements and the institutional needs of all researchers, bespoke templates for the collection of data and metadata for each lab and data type, and secure data storage in dedicated hardware, making project results available internally via web browser, command line, and relational database. All project results were transferred to the InfoCore for validation prior to internal release and analysis. In addition to the quality control requirements used by public repositories, every MaHPIC dataset was internally validated with custom SOPs generated with data producers, scripts to validate essential information about the dataset, and manual inspection of all files and results. SOPs were continuously updated, and member training was repeated as needed based on emerging needs. More detail about InfoCore activities is available in^9^.

### Clinical, haematological, parasitological data

The collection of physical samples and the veterinary care of experimental subjects was key to all downstream results analyses. All physical samples were barcoded and tracked with a Nautilus LIMS managed by Emory University. Automated data collection was used where possible to capture data and metadata for clinical assessments, parasitaemias, animal handing, treatment, diet, snacks, and procedures. Required manual data entry for NHP and human samples were entered into the LIMS or electronic spreadsheets via double-data entry to increase accuracy. Custom collection templates were designed with limits on data entry and pull-down lists of allowable values to further increase accuracy. Both automatically collected information and manual templates and SOPs for subject care are included in public datasets.

### Immune profiling (flow cytometry)

Flow cytometer QC was performed daily by the flow core staff to ensure the instrument was optimally running. Since data was longitudinally compared, voltage control was additionally checked and set by adjusting PMTs just prior to acquiring samples. Each panel had a set of MFI targets per channel for Biolegend 6-peak Rainbow Beads. These beads were run just prior to samples to optimally set PMTs for longitudinal voltage control. They were also used as a QC check to ensure the instrument was optimally performing, as any fluidic or laser problem would result in this check failing. Rainbow beads were also run at the end of sample acquisition to check that MFIs have not shifted over the course of running samples, which would indicate a machine problem.

Samples were acquired on two different LSR-II instruments. If possible, a panel was designated to one instrument and acquired on that same instrument throughout the course of the experiment. If that instrument was down for maintenance, then the other instrument was used as a backup. Both instruments had identical filter sets and were QC’d by flow core staff to be as comparable as possible.

Compensation was performed fresh each day that samples were acquired. The exception was for panels X, Y, and Z due to their compensation requiring cell controls and the limited blood volume available on sample collection days to run these each time. For those panels, compensation was performed at the start of the experiment and applied to each sample within the experiment. If any major instrument changes occurred during the course of the longitudinal experiment, then compensation was rerun.

Stained samples were kept in the dark at 4 °C until the acquisition, which was done within 24hrs.

Antibody lots were tracked during the course of experiments. If possible, antibodies were ordered in bulk to reduce the number of different lots used during the experiment for minimal MFI lot-to-lot variability.

Antibody master mixes were made in bulk for each experimental day to ensure consistency across samples within the same timepoint. Master mixes were either made same day or were stability tested and made within the stability timeframe prior to each timepoint to ensure minimal fluorophore degradation.

Each flow panel was optimized prior to experimental start dates. This included antibody titration, optimal PMT setting, and optimal compensation titration, and bead/cell use decision.

During gating analysis of flow cytometry data, any staining concerns were noted and confirmed by two individuals. These were notated on the data descriptor documents, and if the data was unusable for any days or markers, that was notated, and the data was not included in the statistics files.

### Immune profiling (cytokine)

To reduce batch effects by plate, all samples for Cytokine analysis were randomized across plates. Samples were run in duplicate to ensure consistency. Sample concentrations were calculated based on a standard curve, also run in duplicate. Duplicate blank wells were used to subtract the background.

### Immune profiling (ELISA)

To reduce batch effects by plate, all samples for ELISAs were randomized across plates. Samples were run in duplicate to ensure consistency. Assay was repeated for samples when the CV between duplicates was too high, indicating a technical error in pipetting. Each plate was run with a positive (high) control and a negative (low) control to confirm that results from the plate were acceptable. For assays that reported concentrations, sample concentrations were calculated based on a standard curve which were also run in duplicate. Duplicate blank wells were used to subtract the background.

### Transcriptomics (E13, E04, E04 R, E23R, E24, E25)

The quality of all RNA samples was confirmed using a Bioanalyzer, with an RNA Integrity Number (RIN) greater than 8 recorded for all samples. As a quality control for library preparation, 92 spike-in RNAs of known concentration and GC proportions (ERCC Spike-In Control, Life Technologies) were added to constitute approximately 1% of the total RNA for each library. For E13 and E04, several quality control steps were used to verify the reliability of the base calling and read mapping data: linear correlation of estimated abundance of ERCC spike-in controls with known concentration; confirmation of 99.9% strand-specificity of the controls; less than 0.1% control fusion transcripts; and absence of 3’ bias in the controls was confirmed with RSeqC software (https://code.google.com/p/rseqc). For E04R, E23R, E24, and E25, FASTQC (v0.10.1, https://www.bioinformatics.babraham.ac.uk/projects/fastqc/) was used to assess data quality and Picard Tools (http://broadinstitute.github.io/picard) was used to evaluate mapping quality and 5′-3′ coverage uniformity.

- Note that no spike-ins were used for E24 or E25 samples

### Metabolomics

Quality assurance and results validation were performed as described in the dataset SOPs and in Uppal, *et al*.^25^.

### Lipidomics (E04, E23, E25)

Results were validated via relative quantification. The peak area of each species in a lipid class and their internal standard were integrated by Xcalibur v2.0.7 for data generated on TSQ (Thermo Fisher Scientific) and Analyst v1.5.1 for data generated on API4000 (AB Sciex). The peak area ratio of each species to the internal standard of that lipid class was generated and used as a readout of relative quantification.

$${\rm{Peak}}\;{\rm{area}}\;{\rm{ratio}}=\frac{{\rm{Peak}}\;{\rm{area}}\;{\rm{of}}\;{\rm{lipid}}\;{\rm{species}}}{Peak\;area\;of\;internal\;standard}$$

Only LC-MS/MS runs with an intra-batch CV% < 15% in QC samples (from injection #7 to the last injection) were accepted. Any LC-MS/MS runs with intra-batch CV% > 15% in QC samples were re-run after column or ion source are cleaned. To reduce the effect of inter-batch instrument fluctuation, the peak area ratios of each lipid species in study samples were normalized to the mean of peak area ratios of the same lipid species in the QC sample in the same batch. The QC normalized data can be normalized further with cell count or protein.

$${\rm{Mean}}\;{\rm{of}}\;{\rm{peak}}\;{\rm{area}}\;{\rm{ratio}}\;{\rm{in}}\;{\rm{QC}}(\bar{X}{\rm{QC}})=\frac{{\sum }_{{\rm{i}}=1}^{n}{\rm{individual}}\;{\rm{QC}}\;{\rm{peak}}\;{\rm{area}}\;{\rm{ratio}}(X{\rm{QC}})}{{\rm{number}}\;{\rm{of}}\;{\rm{QC}}\;{\rm{in}}\;{\rm{the}}\;{\rm{same}}\;{\rm{run}}(n)}$$

$${\rm{QC}}\rightleftarrows {\rm{standard}}\,{\rm{deviation}}\,({\rm{SDQC}})=\sqrt{\frac{{\sum }_{i=1}^{n}{[X{\rm{QC}}-\bar{X}{\rm{QC}}]}^{2}}{n-1}}$$

$$ \% {\rm{CV}}=\frac{{\rm{SDQC}}}{\bar{X}{\rm{QC}}}\times 100$$

$${\rm{QC}}\;{\rm{normalized}}\;{\rm{study}}\;{\rm{sample}}\;{\rm{data}}=\frac{Study\;sample\;peak\;area\;ration(X)}{\bar{X}{\rm{QC}}}$$

### Lipidomics (E13)

Retention time and peak integration for each analyte and internal standard were verified for all experimental and calibrator samples. The correlation coefficient for each analyte’s calibration curve was evaluated based on a standard of R^2^ > 0.99. Results for each injected sample include an accuracy measure of the calculated concentration, expressed as a percentage, based on the known concentration of the standards used.

### Proteomics

Quality assurance and results validation were performed as described in the dataset SOPs and in^33^.

## Usage Notes

Publications that make use of data are included as examples of data usage for each experiment. Explanatory README files accompany all datasets for all experiments. These include usage notes distilled from the dataset files and provided by the data-generators. Many of these notes are included below, but *do not substitute for a careful review of the README files for each dataset*. It is difficult to overstate the importance of the README files for reuse of any of these data.

### Experiment 13

#### E13 Publications

Lee *et al*.^8^ presents a multi-omic approach to understanding the effects that pyrimethamine has on immune physiology in rhesus macaques. Whole blood and BM RNA-Seq and plasma metabolome profiles were generated and analyzed for time points before, during and after drug administration.

E13 Clinical, haematological, parasitological data usage notes

- Compared to other experiments, limited metadata is available for the dataset.
- Note E13 blood samples were only analysed for CBC and reticulocyte counts.
- Missing results
  - NHP RZe13 is missing CBC results for day 7/22/13.
  - NHP RTi13 is missing CBC results for day 5/28/13.
  - NHP Rz313 is missing results for reticulocytes on day 7/22/13.

E13 Transcriptomics usage notes

- Index hopping. Results were likely affected by a phenomenon known as ‘Index hopping’. See the README and an Illumina white paper^79^ for technical details.
- Gene-level issues. During mapping of transcript reads to reference genome sequence, the cuffcompare tool used to reformat gff files to gtf caused 146 cases where multiple gene models were collapsed into a single gene model. This affected 328 genes total. See README for full details.
- Exon-level issues. The original reference genome annotation does not contain exon IDs. Exon-level transcript abundance values and exon IDs from the data production core were added to the results template that includes genomic coordinates for each exon. The abundance values themselves were not altered or further processed. For RNA-Seq read mapping, the exon boundaries and membership in genes were modified. Essentially, it is ensured that every genomic location (nucleotide base) belonged to only one exon, and each exon belonged to one and only one gene. Exons shared by multiple genes are listed only once, and their membership is assigned to only one gene (usually, the largest gene spanning the exon). But, the true membership of this exon, including all transcripts and genes that it is a part of is documented in the column “Gene and Transcript Membership of Exon”. In the case of overlapping exons, artificial boundaries are created at the point of overlap so that the nucleotides belong to only one exon. Hence, the number of exons listed, boundaries of exons, and exon IDs are different from the original
- Split and small exons. Due to the adjustment of boundaries, as described above, some exons are split, resulting in exons that are as small as one base long.
  E13 Lipidomics usage notes
- Reference sample. The dataset contains the 7-digit barcode identifiers for each experimental sample. Though sample 2105050 is listed in the analytical metadata sheet as the MaHPIC reference sample barcode, this sample was not, and no data are available.

### Experiments 04 and E04R

#### E04 Publications

Joyner, *et al*.^10^ provides an in-depth analysis of E04 longitudinal clinical and parasitological data. Primary and relapse blood-stage infections were studied. Anaemia and thrombocytopaenia results and inter-individual differences in disease severity are reported. Unlike the primary infections, relapses did not cause clinical malaria. This longitudinal study also produced a variety of datasets from blood and BM samples to support future systems biology investigations.

J. Joyner, *et al*.^11^ details gross pathology and histopathology of the tissues of one rhesus macaque from E04 that developed severe and complicated malaria. The animal had hyperparasitaemia, severe anaemia, and thrombocytopaenia, and despite treatment, demonstrated irreversible renal failure, as well as lung, liver, spleen, and BM abnormalities and specific findings comparable to patients with malaria.

Tang, *et al*.^12^ presents a proof of concept that dynamic models of metabolic pathway systems may be useful for interpreting transcriptomic profiles measured during disease. Transcriptome data for enzymes associated with purine metabolism were analyzed from E04 longitudinal infections, and for comparison from rhesus macaque infections with *P. coatneyi* sporozoites^80^, and applied in a dynamic model of purine metabolism. The model-based interpretation reveals patterns of flux redistribution within the purine pathway, which are also reflected in data from humans infected with *P. falciparum*.

Fonseca, *et al*.^14^ used time-series data to design a mathematical model to identify and quantify a sub-population of *P. cynomolgi* infected RBCs during infection of rhesus monkeys that was non-circulating in the bloodstream (i.e., concealed), and made inferences pointing to similar data for human infections with *P. vivax*. These determinations are critical for understanding *P. vivax* and *P. cynomolgi* biology, pathogenesis, epidemiology, and transmission, as well as treatment strategies.

Fonseca, *et al*.^15^ presents a discrete recursive model with age structure used for longitudinal analyses of *P. cynomolgi* infections in rhesus monkeys to investigate the interplay between the host’s haemodynamic processes and the parasite during *P. cynomolgi* infections with the goal of better understanding *P. vivax* malaria in humans. The study found that an increase in the reticulocyte host-cell subpopulation in the peripheral blood prior to patency is due to the earlier release of younger reticulocytes into the circulation. Additionally, the study determined that loss of infected RBCs only accounted for 38% of the RBC losses due to the infection, while 62% of the RBCs were lost due to a bystander effect.

Tang, *et al*.^81^ integrated multiple data types from BM and peripheral blood to identify dysfunctional BM mechanisms during the E04 longitudinal *P. cynomolgi*-rhesus monkey infections. This work revealed significant alteration in the BM transcriptome, and inflammation pathways were enriched. Analysis of the cell types involved pointed to changes in erythroid progenitors, the possibility that GATA1/GATA2 master regulators of erythroid differentiation may be disrupted, and that monocyte-driven inflammation could suppress erythropoiesis *in vivo*.

E04 Clinical, haematological, parasitological data usage notes

- Final results for parasitaemia data. For this experiment, a third parasite reader was used. To avoid potential confusion from the use of three parasitaemia readers, the data fields “Parasites/µL FINAL” and “% Parasitemia FINAL” are included in the consolidated results file.
- Manual data entry. Due to the infrastructure and method of data collection available for E04, manual data entry was inevitable. While personnel took several measures to guard against manual data entry errors, users of these data should bear this in mind. Among the measures employed: two microscopists read the parasitaemia slides independently and recorded their results separately. Data entered into the LIMS were double-checked by comparing against printed copies of results. Several lab meetings were held to review the experiment activities file that was assembled over the course of this ~100-day experiment.
- Experiment “activities” file. This file was manually assembled throughout the course of the experiment, from various sources of observations of activities and events. Along with the “major” events such as days of treatment, and determination of TP days, several other events are also documented. Examples of such events include an NHP cutting its foot, sedation on a non-TP collection day, etc. Every attempt was made to generate a comprehensive document with observations and notes throughout the experiment. However, users should be aware that some irregularities may remain. For example, cage washes were noted in the file when the personnel extracting the samples observed this event. Cage washes that were not directly observed by these personnel are not available in this experiment activities summary file.
- Blood transfusion events. Two NHP subjects - RMe14 and RFv13 - received blood transfusions, from different donor monkeys. RMe14 received a transfusion from NHP with ID CG80, and RFv13 received a transfusion from NHP with ID CF97. These transfusions occurred on day 22 of the experiment. No samples were collected from donor CG80. Blood samples were collected from donor CF97, but 41 days after the transfusion. Clinical data were not generated for this donor sample.
- Euthanasia events. RFv13 was euthanised on 09/28/2013 due to clinical complications of infection^11^.
- Lack of data for some parasitaemias. For the thick blood smear parasitaemia readings, cells have the value “Not entered” when the parasitaemias were over 1%, and the procedure lost accuracy.
- Only the CBC data were captured from the haematology analyser. Manual counts were conducted for reticulocytes, thin and thick blood smears, and normoblast.

E04 Immune profiling usage notes (ELISA assay)

- All samples were run in duplicate and randomized on the plate. Note that for the raw results, values presented are for the average of the two technical replicates, so columns F, G, H, I, and J are blank for one member of each replicate pair.
- Different from most MaHPIC datasets, the raw and final results are combined as separate excel worksheets in the same workbook.
- Results were calculated based on a standard curve. See dataset SOPs for more information.

E04, E04R Transcriptomic usage notes

- For both E04 and E04R there are separate README files for the sequence generation and mapping. Unless specified, the notes below apply to both E04 and E04R.
- Control samples. E04 transcriptomic datasets do not include samples from the control NHP REe6. In contrast, E04R transcriptomic datasets do include samples from this control animal.
- Index hopping. Results were likely affected by a phenomenon known as ‘Index hopping’. See the README and an Illumina white paper^79^ for technical details.
- Two E04 WB samples failed library preparation and were not sequenced. NHP RSb14, TP5 and NHP RFv13, TP2.
- E04 Gene-level issues. During mapping of transcript reads to reference genome sequence, the cuffcompare tool used to reformat gff files to gtf caused 146 cases where multiple gene models were collapsed into a single gene model. This affected 328 genes total. See READMEs for full details.
- E04 Exon mapping. While these results were initially planned and are referenced in the README they are not included in the dataset and are no longer planned for release.
- E04R Additional sequencing depth.
  - Due to a technical issue with the flowcell of the first sequencing run, the output of the run did not produce the desired read depth and so E04R samples were sequenced a second time on a second flowcell.
  - E04R TP2 samples were sequenced at additional depth to account for dominance of parasite reads at that TP.
  - E04R read files from both runs were aligned together to generate the BAM files, raw read count tables, and Coverage QC files.

- E04R Mapping results counts.
  - Reference Genome Annotation Files and Results Counts Files - The mapping SOP generated abundance measures for each unique gene ID in the reference genome annotation file. This is implemented in the STAR alignment tool using the HTSeq-count algorithm. For the genes with multiple transcript models, reads that mapped uniquely to any exon of any alternate transcripts were counted once for the gene ID of those transcripts. They are not ‘binned’ together with reads that mapped to multiple loci, or with ambiguously mapped reads. Essentially, expression for alternate transcripts of a single gene were accumulated together.
  - If the host reference genome annotation file contained different gene names (shown as gene symbols in the Results Counts file(s)) with the same gene IDs, the mapping SOP assigned the last gene name encountered in the annotation file to the corresponding gene ID in the Results Counts file(s). There are 2 instances (gene IDs 653149 and 6884) where the same gene ID had multiple corresponding gene names. Each different gene name corresponds to a unique locus with the same gene ID. Reads that mapped to unique loci with the same gene ID were binned together for that gene ID. However, the loci may be highly similar, and reads that map to multiple locations may have been binned as ambiguous and not counted for any gene ID.
  - The supporting clinical information file for E04R is the same as for E04 since both are based on the same NHPs and clinical progression of disease. The file name indicates E04 for both.
  - The analytical metadata and raw counts files include references to ‘E04’ and not ‘E04R’. This is due to how samples were handled internally at the sequencing center that generated them. The file names indicate the correct experiment.

E04 Metabolomics usage notes

- Computational annotations. There are multiple-to-multiple matches between ions (mz + time combos) and the possible chemical IDs. This is the state of the field (at the time of the analyses).
- Results from C18 positive and C18 negative modes may not be combined. Intensity level is relative within each ionization mode and cannot be compared between the modes.
- There are no expectations of overlap between positive and negative modes of C18.
- Finding ions is not guaranteed in all three replicates of a sample. Ion intensity value for a particular ion (mz + time combo) may be zero in two of the three replicate samples. Treat zeros as missing values and not as zero values, and do not average the intensities across the triplicates.

E04 Lipidomics usage notes

- Normalized results

- All users should prefer using the files which contain QC based normalization results.
- The data should be further normalized based on other parameters such as the RBC counts and WBC counts. Relevant data are provided as separate files, extracted from E04 Clinical data.

- Limitations on sphingolipid detection. ST and GMD are two classes of sphingolipids that are low abundant, and TSQ cannot be used for those.
- Analysis runs. There were 4 runs. The first 3 runs were for the abundant sphingolipids and phospholipids and were run on TSQ. The fourth run was for the low abundant sphingolipids, and API4000 was used.
- Intermediate results. These data are provided for each lipid species in separate files. They contain the peak area and other data. These have not been QC-normalized.

E04 Proteomics usage notes

- In the primary results file, negative values (−1, −2, −3) have special meanings defined in the column header definitions and do not represent real data values.

−3 = Not present in either TP1 or the experimental TP of that NHP subject
−2 = Not present in the experimental, but present in TP1 of that NHP subject
−1 = Present in the experimental but not in TP1 of that NHP subject

### Experiment 23

#### E23 Publications

Joyner, *et al*.^18^ provides an in-depth analysis of E23 and E24 longitudinal clinical and parasitological data. Primary and relapse blood-stage infections were studied after a primary infection with *P. cynomolgi* M/B strain sporozoites (E23) and homologous challenge with *P. cynomolgi* M/B strain sporozoites (E24)^1^. Anaemia and thrombocytopaenia results and inter-individual differences in disease severity are reported. Unlike the primary infections, neither relapses nor homologous reinfections caused clinical malaria. Clinical immunity, including memory B cells, developed after a single sporozoite-initiated blood-stage infection. Rapid memory B cell recall responses were observed, with anti-parasite IgG1 activity that helped to reduce the asexual stage parasitaemia. While the total number of circulating gametocytes was reduced, the cumulative proportion of gametocytes increased during relapses. These experiments also produced a variety of datasets from blood and BM samples to support future systems biology investigations.

E23R Clinical, haematological, parasitological data usage notes

- Unequal number of TP collections for the six NHPs. Time points were designated as described above. Based on this structure, and due to the kinetics of the infection, it was possible to identify peak infection or relapse for some animals, and samples were collected accordingly. For other animals, infection kinetics were difficult to predict. Specifically:

- RIb13 and RBg14 do not have TP7 collections.
- None of the NHPs have shown a second relapse. Hence, there are no TP6 collections.
- ROh14 did not show indications of a relapse after undergoing a blood transfusion.

- Blood transfusion events. On Day 22, subject ROh14 was given blood transfusion – 100 ml – from donor CF9D. No samples were collected from donor CF9D.
- Sample collection and data entry issues. There were several notable issues with sample collection and storage. See the README for details.
- Parasitaemia calculation. Parasitaemia counts were determined by two microscopists by reading the slides independently. A third microscopist was employed when the parasitaemia was high or when the readings by the first and second reader varied widely. The average of the closest two readings was reported as the final parasitaemia. A file with the schematic representing this calculation is available and listed elsewhere in this file, and also included on a separate sheet in the consolidated results file.
- Corrections performed. When normoblasts (nucleated, immature RBCs) are occasionally present in peripheral blood, the instrument reads them as monocytes or lymphocytes. Thin blood smears are scanned for the presence of nucleated RBCs. If they are detected, differential cell counts are performed. WBCs are corrected to adjust for the nucleated RBC counts. Essential, agreed upon, corrections to the data were performed and documented in a supporting document: E23M99MEMmCyDaWB_Corrections-Done_MULTIPL.docx

E23 Immune profiling usage notes (flow cytometry)

- As opposed to the panels C & D in E04, E24, and E25, which used WB, panels C and D in E23 used PB.
- There are no results from TP01 for many panels. There are no results from TP1A for panels C and D. This means that analytical conclusions are limited since you cannot compare to baseline but can still compare a specific TP to other data types.
- For some NHPs TPs overlapped. This resulted in fcs files from different TPs being organized into the same folders. File and folder names and the analytical metadata can be used to clarify. Examples include TP3A, TP04 and TP05.
- Gating images for panels X, Y, and Z are identical across E23, E24, and E25 because the gating strategy was the same for all of these experiments.
- No calibration fcs files are available for the folder “E23T3AAIMMCYXXZZ_RJn13” due to a file export error at the cytometer. Calibration was performed prior to running samples.
- In addition to the panels described in the methods section, there are a number of additional panels that did not produce reportable final results. Unless otherwise noted, results are not available for the following panels:
  - entire panels K, V, O, P, Q, R
  - TP1 samples NR for panels I, J, N, L, M, C, D
  - TP1A samples NR or panels C, D
  - TP7 samples NR for panels I, J
  - panel M, any results using the staining marker ccr7
- For panel M all results using marker ccr7 are NR and were removed from the final results.
  E23 Immune profiling usage notes (cytokine assay)
- E23, E24, and E25 immunology cytokine results were processed together. Samples were randomized across 3 plates; due to an error loading plate 2, standards in plate 2 are in different well locations than plate 1 or plate 3.
- No TP1A samples. TP1A samples were excluded from E23 data production because of limited available plasma.
- Values of “0” occur in the finalized results. A value of 0 when present should be taken to mean a value that is below the level of detection for a particular cytokine. The lower level of detection is different for each cytokine, based on its standard curve. This information can be found in the ProcartaPlex Analysis pdfs, which include additional information about the standard curve for each cytokine based on which plate the sample of interest was run.
- Minor differences from E04, a preceding iteration of this experiment. E23, E24, and E25 samples are from cryopreserved plasma - E23, E24, E25 results are derived from undiluted venous PS, that was cryopreserved at −80 C, prepared without Lymphoprep. This differs from diluted CR prepared with Lymphoprep in E04. The manufacturer producing the custom kit for analysing these 45 cytokines changed hands between E04 and E23, but catalogue numbers noted in the Analytical metadata remain unchanged and identical. E23, E24, and E25 data were collected using a more recent version of the same acquisition software used in E04.
  E23 Immune profiling usage notes (ELISA assays)
- Samples from E23, E24, and E25 were randomized across all experiments, processed, and analysed together.
- Depending on the number of samples in an assay, samples were in up to three plates. Additionally, each plate was run in two replicates.
- In some cases, an entire plate needed to be repeated, or just some samples needed to be repeated; in these cases, there are additional sheets in the raw result files. See data integrity sections of the SOPs for reasons why repeats may have been needed. When a result is from a repeat sample, it is indicated with “red” colour in the combined result file to bring it to the consumer’s attention.
- Results were calculated based on a standard curve. See dataset SOPs for more information. Note that since standards were not available for the IgG subclasses (IgG1, IgG2, IgG3), a relative measure, Optical Density (OD), was reported instead.
- Not all samples were run & analysed in every assay. See the below matrix for details.
  - Total IgG, Total IgM, iRBC IgG – M/B strain, uRBC IgG – M/B strain, iRBC IgG1 – M/B strain, iRBC IgG2 – M/B strain, iRBC IgG3 – M/B strain: All samples were run & analysed. Total samples analysed: 88.
  - iRBC IgM – M/B strain, uRBC IgM – M/B strain, iRBC IgM - Ceylon strain, uRBC IgM - Ceylon strain: T01 samples from E23 and T07 samples from E25 were not analysed. Total samples analysed: 77
  - iRBC IgG1 – Ceylon strain, iRBC IgG2 – Ceylon strain, iRBC IgG3 – Ceylon strain: Only samples from E23 T01, E23 T05, E24 T01, E24 T03, E25 T01, E25 T1B, E25 T02, and E25 T03 were run and analysed. Total samples analysed: 41
  - iRBC IgG – Ceylon strain, uRBC IgG – Ceylon strain: None of the E23 samples except T05 and none of the E24 samples except T03 were analysed. No RJn13 samples from E23 were analysed. Total samples analysed: 35

- Blood transfusion (see E23 clinical usage notes). This is expected to impact many events for up to 100 or 120 days. Use data from this subject post-transfusion with caution.
- Use QC normalized data for analyses is recommended.
- Data should be further normalized by RBC and/or WBC counts. RBC and WBC counts data are available in a separate file.
- Four lipid species failed the QC check: PC(P18:3-18:1), DG(16:0-18:2) and DG(18:1-16:0) from BM, and GC(24:0) from WB.

E23R Transcriptomic usage notes

- There are separate README files for the sequence generation and mapping.
- Homologous/heterologous challenges. E23R transcriptomics data is intended for comparison with data from E04R, E24, and E25.
- Sequencing. All samples were run in 7 lanes, produced 14 read files, at 50 M reads/sample with the exception of TP2 samples, which were run in 8 lanes, produced 16 read files, at 100 M reads/sample.
- Sequencing. One sample from TP1b (2621369) was sequenced twice due to library prep processing failure. See the README file for details.
- Reference genome annotation files and results counts files.
  - The mapping SOP generated abundance measures for each unique gene ID in the reference genome annotation file. This is implemented in the STAR alignment tool using the Htseq-count algorithm. For genes with multiple transcript models, reads that map uniquely to any exon of any alternate transcripts are counted once for the gene ID of those transcripts. They are not ‘binned’ together with reads that map to multiple loci, or with ambiguously mapped reads. Essentially, expression reads for alternate transcripts of a single gene are accumulated together.
  - If the host reference genome annotation file contained different gene names (shown as gene symbols in the Results Counts file(s)) with the same gene IDs, the mapping SOP assigns the last gene name encountered in the annotation file to the corresponding gene ID in the Results Counts file(s). In the host annotation file, there are 2 instances (gene IDs 653149 and 6884) where the same gene ID has multiple corresponding gene names. Each different gene name corresponds to a unique locus with the same gene ID. Reads that map to unique loci with the same gene ID will be binned together for that gene ID. However, the loci may be highly similar and reads that to multiple locations will be binned as ambiguous and not counted for any gene ID.

- Index hopping. Results were likely affected by a phenomenon known as ‘Index hopping’. See the README and an Illumina white paper^79^ for technical details.

E23 Lipidomics usage notes

- Use of QC normalized data for analyses is recommended.
- Data should be further normalized by RBC and/or WBC counts. RBC and WBC counts data are available in a separate file.
- 4 lipid species failed QC check: PC(P18:3-18:1), DG(16:0-18:2) and DG(18:1-16:0) from BM, and GC(24:0) from WB.

E23 Proteomics usage notes

- Results definitions. In the results file E23M99PRMmCyDaRC_Results_MULTIPL.xlsx note that negative values (−1, −2, −3) have special meanings defined in the column header definitions and do not represent real data values (see section 4. Experimental Results Column Header Definitions).
- NHP subject ROc14. This animal was previously used in MaHPIC Control Experiment 18.

### Experiment 24

Joyner, *et al*.^18^ provides an in-depth analysis of E23 and E24 longitudinal clinical and parasitological data. Primary and relapse blood-stage infections were studied after a primary infection with *P. cynomolgi* M/B strain sporozoites (E23) and homologous challenge with *P. cynomolgi* M/B strain sporozoites (E24)^1^. Anaemia and thrombocytopaenia results and inter-individual differences in disease severity are reported. Unlike the primary infections, neither relapses nor homologous reinfections caused clinical malaria. Clinical immunity, including memory B cells, developed after a single sporozoite-initiated blood-stage infection. Rapid memory B cell recall responses were observed, with anti-parasite IgG1 activity that helped to reduce the asexual stage parasitaemia. While the total number of circulating gametocytes was reduced, the cumulative proportion of gametocytes increased during relapses. These experiments also produced a variety of datasets from blood and BM samples to support future systems biology investigations.

E24 Clinical, haematological, parasitological data usage notes

- Subject RJn13 was dropped from the study after E23. Due to persistent behavioral issues and animal inaccessibility for sample collection.
- There is no TP2. The name “Time Point 3” was used to indicate the post-peak of infection because the peak was missed; the NHPs controlled/self-resolved the hyper-parasitemia. Artemether was planned to be used during this period, but it was not used because the animals self-resolved.
- Parasitaemia calculation. Parasitaemia counts were determined by two microscopists by reading the blood smear slides independently. A third microscopist was employed when the parasitaemia was high or when the readings by the first and second reader occasionally varied widely. The average of the closest two readings was reported as the final parasitaemia. See the SOP for details: E24_E25_Malaria_Core_SOP_V10_July2016_FINAL.docx
- Corrections performed. When normoblasts (nucleated immature RBCs) are present in the peripheral blood, the instrument reads them as monocytes or lymphocytes. Thin blood smears are scanned for the presence of nucleated RBCs. If they are detected, differential cell counts are performed. WBCs are corrected to adjust for the nucleated RBC counts. Essential, agreed upon, corrections to the data were performed and documented in supporting materials in the public dataset.

E24 Immune profiling usage notes (flow cytometry)

- Gating images for panels X, Y, and Z are identical across E23, E24, and E25 because the gating strategy was the same for all experiments.
- For panels X, Y, Z E23 compensation files were used to create compensation matrices (see specifically the contents of folder E23M99IIMMCYXXZZ_COMPS_PANELZ_PANELY_PANELX in E23 raw files). Only the compensation matrices are present in the E24 raw results. See the E23 Immunology dataset for the compensation fcs files used to create these matrices.
- In addition to the panels described in the methods section, there are a number of additional panels that did not produce reportable final results. Unless otherwise noted, results are not available for the following panels:
  - entire panels AF, V, O, T, U, AB, AC, P, Q, AG
  - TP1B PB samples for NHPs Rad14 and Rlb13 panels S, AA
- Analytical metadata contain worksheets for individual sample collection days. At the bottom of each worksheet are notes from MaHPIC’s Immunology core that detail which NHPs had samples processed on that day and any issues with sample collection or processing.
  E24 Immune profiling usage notes (cytokine assay)
- Samples from E23, E24, and E25 were randomized across all experiments, processed, and analysed together. See usage notes for E23 Cytokine dataset as they apply to this dataset as well.
  E24 Immune profiling usage notes (ELISA assays)
- Samples from E23, E24, and E25 were randomized across all experiments, processed, and analysed together. See usage notes for E23 ELISA dataset as they apply to this dataset as well.

E24 Transcriptomic usage notes

- There are separate README files for the sequence generation and mapping.
- Homologous challenge. E24 transcriptomics data is intended for comparison with data from E04R, E23R, and E25.
- ERCC spike-ins were not included as a part of this dataset.
- Reference genome annotation files and results counts files. See usage notes for E23R.
- Index hopping. Results were likely affected by a phenomenon known as ‘Index hopping’. See the README and an Illumina white paper^79^ for technical details.

### Experiment 25

#### E25 Summary

E25 includes an in-depth analysis of longitudinal clinical and parasitological data resulting from the heterologous challenge of animals for E24^1^, Joyner, *et al*.^18^. Unlike with the homologous reinfection experiment (E24), protection was not achieved with a subsequent heterologous reinfection (with *P. cynomolgi* Chesson strain sporozoites).

E25 Clinical, haematological, parasitological data usage notes

- Subject RJn13 was dropped from the study after E23, due to persistent behavioural issues and animal inaccessibility for sample collection.
- Subject ROh14 evidenced a relapse with M/B strain. Parasites were observed on the thick blood film for ROh14 six days after the animal was inoculated with *P. cynomolgi* Ceylon strain sporozoites. The liver stage of *P. cynomolgi* is approximately 8 days, and blood-stage parasites are not typically observed until Day 11 or Day 12 after inoculation with 2,000 sporozoites per the protocol used for E04, E23, E24, and E25. The appearance of infected RBCs by Day 6 prompted investigation of whether the radical cure following E24 did not eliminate all blood and/or liver-stage parasites. To test this hypothesis, blood pellets collected during ROh14’s infection were sent to the CDC, and the circumsporozoite (CSP) gene was amplified via PCR and sequenced. The sequence of the CSP gene revealed that these parasites were not *P. cynomolgi* Ceylon, but they were *P. cynomolgi* M/B strain parasites^82^, which had been used in E23 and E24. It was concluded that the parasites observed on Day 6 were *P. cynomolgi* M/B strain due to hypnozoites that activated and caused another relapsing blood-stage infection. Taking these circumstances into account, ROh14 should be excluded from most E25 analyses since the other animals did not experience a relapse with the previous strain.
- Parasitaemia calculation. Parasitaemia counts were determined by two microscopists by reading the slides independently. A third microscopist was employed when the parasitaemia was high or when the readings by the first and second reader occasionally varied widely. The average of the closest two readings was reported as the final parasitaemia. For details regarding the rules for use of thick and thin blood smears for these calculations, please refer to the SOP: E24_E25_Malaria_Core_SOP_V10_July2016_FINAL.docx.
- Corrections performed. When normoblasts (nucleated immature RBCs) are present in the peripheral blood, the instrument reads them as monocytes or lymphocytes. Thin blood smears are scanned for the presence of nucleated RBCs. If they are detected, differential cell counts are performed. WBCs are corrected to adjust for the nucleated RBC counts. Essential, agreed upon, corrections to the data were performed and documented in supporting materials in the public dataset.
- Critical information is available in the daily activities file. Examples of critical information pertinent to some analyses include days when a different haematology analyser was used, any special medication needs for a given NHP, etc.

E25 Immune profiling usage notes (flow cytometry)

- Panels S and AA were switched back to PB due to inconsistent staining of WB at baseline. This is different from E24 where WB was used.
- Gating images for panels X, Y, and Z are identical across E23, E24, and E25 because the gating strategy was the same for all experiments.
- For panels X, Y, Z E23 compensation files were used to create compensation matrices (see specifically the contents of folder E23M99IIMMCYXXZZ_COMPS_PANELZ_PANELY_PANELX in E23 raw files). Only the compensation matrices are present in the E24 raw results. See the E23 Immunology dataset for the compensation fcs files used to create these matrices.
- In addition to the panels described in the methods section, there are additional panels that did not produce reportable final results. Unless otherwise noted, results are not available for the following panels:
  - entire panels entire panels K, V, T, O, U, AB, AC, P, Q, AG, AF
  - TP1A panels AD, AE
  - TP01 WB sample panels S, AA
- Analytical metadata contain worksheets for individual sample collection days. At the bottom of each worksheet are notes from the MaHPIC Immunology core that detail which NHPs had samples processed on a given day and any issues with sample collection or processing.
  E25 Immune profiling usage notes (cytokine assay)
- Samples from E23, E24, and E25 were randomized across all experiments, processed, and analysed together. See usage notes for E23 Cytokine dataset as they apply to this dataset as well.
  E25 Immune profiling usage notes (ELISA assays)
- Samples from E23, E24, and E25 were randomized across all experiments, processed, and analysed together. See usage notes for E23 ELISA dataset as they apply to this dataset as well.

E25 Transcriptomic usage notes

- There are separate README files for the sequence generation and mapping.
- Heterologous challenge. E25 transcriptomics data is intended for comparison with data from E04R, E23R, and E24.
- Parasite reference. *P. cynomolgi* Ceylon strain was used to infect NHPs, but due to reference genome unavailability at the time of data production, the *P. cynomolgi* M/B strain reference genome was used to generate these data.
- ERCC spike-ins were not included as a part of this dataset.
- Reference genome annotation files and results counts files. - see usage notes for E23R
- Index hopping. Results were likely affected by a phenomenon known as ‘Index hopping’. See the README and an Illumina white paper^79^ for technical details.

E25 Lipidomics usage notes

- Use of QC normalized data for analyses is recommended.
- Data should be further normalized by RBC and/or WBC counts. RBC and WBC counts data are available in a separate file.
- 2 lipid species failed QC check: PI(18:0-22:4) and PI(18:0-18:0) from WB.

## Supplementary information

Supplementary Table 1

Supplementary Table 2

Supplementary Table 3

Supplementary Table 4

Supplementary Table 5

## Data Availability

Software used for data generation and analyses are described in detail in the methods sections for each data type and in the references cited in those sections. The project utilized iRODS (https://irods.org) for data storage and organization and an Oracle relational database for supporting mathematical modeling and deposition of data to public repositories. Our relational database implemented the Scientific Knowledge Extraction from Data (SKED) schema^83,84^. An in-house custom tool, xport4sub, was developed to help prepare submissions to pubic repositories. Each public repository’s submission requirements and templates were unique. xport4sub was designed to be configured for a specific repository’s submission templates and to populate those templates automatically from MaHPIC’s relational database. Both the xport4sub tool and the SKED schema are published under MIT license and are available on MaHPIC Github repository (https://github.com/mahpic/).
